# Fighting fire with fire: Prebunking with the use of a plausible meta‐conspiracy framing

**DOI:** 10.1111/bjop.70023

**Published:** 2025-09-08

**Authors:** Mikey Biddlestone, Ricky Green, Daniel Toribio‐Flórez, Dylan de Gourville, Robbie M. Sutton, Karen M. Douglas

**Affiliations:** ^1^ School of Psychology University of Kent Canterbury UK

**Keywords:** climate change, conspiracy beliefs, COVID‐19, misinformation, prebunking

## Abstract

Prebunking can be used to pre‐emptively refute conspiracy narratives. We developed a new approach to prebunking – *fighting fire with fire* – which introduces a plausible ‘meta‐conspiracy’ suggesting that conspiracy theories are deliberately spread as part of a wider conspiracy. In two preregistered intervention studies, prebunking specific COVID‐19 vaccine (Study 1, *N* = 720) and climate change (Study 2, *N* = 1077) conspiracy theories (e.g. that climate change is a hoax), with or without this meta‐conspiracy framing, did not reduce beliefs in these specific conspiracy theories. However, some notable findings emerged. First, both *fighting fire with fire* and standard prebunking (Study 2) increased belief in plausible meta‐conspiracies that questioned the original specific conspiracy theories. Second, across both studies, specific conspiracy beliefs negatively predicted behavioural intentions, while beliefs in meta‐conspiracies positively predicted them. Third, specific conspiracy beliefs were negatively related to belief in plausible meta‐conspiracies in both intervention studies (cf: Pilot Study). While this approach did not reduce specific conspiracy beliefs, it increased beliefs that were negatively associated with them and which were positively linked to behavioural intentions. We discuss these null effects and their implications for effective prebunking among conspiracy believers.

Evidence suggests that climate change denial narratives and COVID‐19 misinformation have been actively manufactured and amplified through coordinated, well‐funded campaigns that aim to undermine public trust in science. In the context of climate change, fossil fuel interests and conservative think tanks have spent decades strategically spreading misinformation to delay policy action and portray scientists as conspiratorial actors (Coan et al., 2021; Lewandowsky, 2021). Similarly, during the COVID‐19 pandemic, just 12 individuals – the so‐called ‘Disinformation Dozen’ – were responsible for nearly two‐thirds of COVID‐19 misinformation on social media, using platforms like Twitter and YouTube to disseminate conspiracy theories and sow distrust toward public health efforts (Nogara et al., 2022). Ironically, a real conspiracy lies in the deliberate spread of conspiracy theories. In this context, we tested whether exposure to a *meta‐conspiracy theory* – the idea that conspiracy theories themselves may be strategic conspiratorial tools used to manipulate the public – could reduce belief in conspiracy narratives. By highlighting the orchestrated nature of climate and COVID‐19 misinformation, we aimed to inoculate individuals against the broader ecosystem of disinformation.

A *meta‐conspiracy theory*, or second‐order conspiracy theory, suggests that conspiracy theories themselves are deliberately spread for ulterior motives (Stamatiadis‐Bréhier, 2024). Independent evidence from *America Misled* (Cook et al., 2019) supports this idea, showing that the fossil fuel industry used tactics from Big Tobacco – cherry‐picking data, promoting fake experts and spreading conspiracy theories – to undermine climate science. Awareness of such meta‐conspiracies may sow doubt about original conspiracy theories by offering an alternative explanation. Inspired by this, we propose that exposure to *plausible* meta‐conspiracies (i.e. meta‐conspiracies that people tend to perceive as relatively more believable than other conspiracy narratives and which hold a great deal of supporting evidence from a variety of different falsifiable and transparent sources; see Dentith, 2022; Hattersley et al., 2022) could serve as interventions to reduce conspiracy endorsement. For instance, if people learn that fossil fuel companies deliberately spread climate denial, they may be less likely to believe that climate change is a hoax. Additionally, since climate conspiracy beliefs have worrying consequences, such as deterring political support for pro‐climate policies (see Biddlestone et al., 2022), introducing plausible meta‐conspiracies could help neutralise this influence by exposing the endless epistemic loop of conspiracist thinking.

This article tests whether introducing a plausible meta‐conspiracy theory – *fighting fire with fire* – can reduce the appeal of specific conspiracy theories by framing them as part of a broader effort to mislead the public. We conducted one correlational pilot study and two intervention experiments to examine whether this approach increases belief in plausible meta‐conspiracies, reduces conspiracy beliefs, and boosts relevant behavioural intentions (e.g. vaccine acceptance, reducing one's carbon footprint). Aligned with this special issue on misinformation in environmental crises, we aim to enhance psychological interventions, particularly for resistant populations who may be trapped in a closed epistemic loop of conspiracy thinking (Sutton & Douglas, 2022). While inoculation is often conceived as a prophylactic intervention (i.e. administered before beliefs form), emerging evidence suggests that it can also serve a therapeutic function – reducing existing conspiracy beliefs and fostering harm reduction among high‐belief individuals (Mason et al., 2024). Our work contributes to this growing literature by testing whether meta‐conspiracy reframing can act as a form of therapeutic inoculation, shifting beliefs even among those already disposed toward conspiracist reasoning.

## Belief in conspiracy theories and interventions to limit their consequences

Conspiracy theories are *epistemically risky* claims that two or more individuals have coordinated in secret to achieve forbidden acts that are of public interest, but not yet public knowledge (Douglas & Sutton, 2023). Examples include the belief that climate change is a hoax or that pharmaceutical companies conceal vaccine harms. Meta‐analyses show such beliefs have negative consequences. Climate conspiracy beliefs reduce pro‐environmental engagement (Biddlestone et al., 2022), while COVID‐19 conspiracy beliefs lowered compliance with health guidelines (Bierwiaczonek et al., 2022) and vaccine acceptance (Stasielowicz, 2022). Conspiracy thinking is also linked to vaccine hesitancy, radicalization and political polarization (Douglas & Sutton, 2023). While not all conspiracy theories are harmful, limiting the impact of those that are is crucial.

Recent reviews and meta‐analyses have begun to quantify the effectiveness of interventions aimed at reducing conspiracy beliefs (O'Mahony et al., 2023; Stasielowicz, 2024). While some promising strategies have emerged, the overall picture remains modest and mixed. Reflective thinking prompts and analytic reasoning have shown limited or context‐dependent effects (Bago et al., 2022; Većkalov et al., 2024). Normative appeals, such as highlighting majority views, may have persuasive potential, but existing work is largely limited to specific populations (Cookson et al., 2021). Fact‐checking has shown small effects overall, and providing alternative explanations has not consistently yielded significant benefits (Chan & Albarracín, 2023; Stasielowicz, 2024). This landscape of limited efficacy underscores the need for new approaches.

Psychological inoculation, or *prebunking*, modestly improves credibility discernment between reliable information and misinformation (*d* = 0.20; Lu et al., 2023), which is defined as deceptive or misleading content contradicting the best available evidence (Southwell et al., 2022). Psychological inoculation pre‐emptively refutes psychological states or manipulation techniques that foster misinformation susceptibility (Traberg et al., 2023). For example, prebunking games like *Bad News* let players adopt the role of a misinformation spreader, using tactics such as conspiracy theories (Basol et al., 2020, 2021; Neylan et al., 2023). While its effectiveness is less clear in non‐WEIRD contexts (e.g. Harjani et al., 2023) or when immediate feedback on learning effects is not provided (Capewell et al., 2023; Leder et al., 2023), most evidence supports prebunking as a useful tool to enhance people's ability to discern manipulative information online (Lu et al., 2023).

O'Mahony et al.'s (2023) recent systematic review also found that inoculation consistently reduces conspiracy beliefs. However, its effectiveness appears to vary by domain. For instance, Spampatti, Hahnel, et al. (2023) observed limited effects of inoculation against climate misinformation, possibly due to the entrenched nature of climate attitudes. However, other research suggests that detailed explanatory interventions, such as offering in‐depth information about COVID‐19 vaccines, can successfully reduce conspiracy beliefs and vaccine hesitancy by offering plausible alternative explanations and reducing uncertainty (Pummerer et al., 2022). Taken together, these findings suggest that inoculation may be most effective when it combines psychological warnings with clear, alternative narratives that address people's informational needs and anxieties.

O'Mahony et al.'s (2023) review highlights inoculation's promise in reducing conspiracy beliefs. Inoculation messages not only mitigate misinformation susceptibility but also improve behavioural intentions otherwise weakened by misinformation, such as vaccine acceptance (Jolley & Douglas, 2017) and voting for pro‐environmental policies (Spampatti, Brosch, et al., 2023). While most interventions aim to reduce belief in conspiracy theories, others have targeted the consequences of such beliefs – such as political disengagement, rejection of science, or opposition to societal initiatives. For example, Winter et al. (2022) found that people with stronger conspiracy mentalities were significantly more likely to oppose the construction of wind farms. Nevertheless, a brief communication emphasising the social and environmental benefits of wind energy could still improve their willingness to support such initiatives – even among those with strong prior conspiracy beliefs. This aligns with the idea that interventions do not always need to eliminate conspiracy beliefs to be effective; they can attenuate their behavioural consequences by reframing the narrative or boosting alternative motivations. Building on this, we explored whether our *meta‐conspiracy* intervention might not only reduce conspiracy beliefs but also improve behavioural intentions – specifically, willingness to receive the COVID‐19 vaccine and to reduce one's carbon footprint.

The idea of warning people that manipulative individuals deliberately spread false information is not new to inoculation messaging (e.g. Basol et al., 2020). In fact, highlighting such actors is key to motivating psychological defences against persuasion (Eccles et al., 2021). Our intervention builds on this by introducing *meta‐conspiracies* – relatively plausible (compared to most other conspiracy theories) secretive efforts to spread misinformation for nefarious purposes. While standard prebunking messages warn about manipulation, we explicitly frame this manipulation in conspiratorial terms. For instance, instead of simply advising vigilance against misinformation, a *fighting fire with fire* prebunking message might say ‘*Remember to question whether the real conspiracy comes from those promoting conspiracy theories*’.

## Meta‐conspiracy theories

Douglas and Sutton (2015) first referenced *meta‐conspiracy theories* when distinguishing between conspiracist claims that sow doubt (e.g. *climate change is a hoax*) and those that support scientific claims. While conspiracy theories allege that governments or scientists deceive the public about climate change, meta‐conspiracy theories suggest that such claims are themselves part of a conspiracy to undermine climate science for financial gain (Hattersley et al., 2022). Stamatiadis‐Bréhier (2024) expanded on this idea, calling them *second‐order conspiracies* and arguing for a *genealogical approach* to debunking conspiracy theories (i.e. addressing the origins of these beliefs instead of the symptomatic beliefs they produce). This genealogical approach suggests that instead of refuting specific conspiracy claims, meta‐conspiracies undermine the origins and motives behind them, making it more difficult for conspiracy theorists to dismiss these refutations as part of the conspiracy.

While conspiracy theories are often endorsed by alternative sources that challenge mainstream views (Pierre, 2020), the *meta‐conspiracy theories* we examine here typically refer to well‐documented cases of conspiracies to spread misinformation, such as The Kremlin's disinformation efforts (Emmott, 2021). Unlike conspiracy theories, which oppose official explanations (Douglas & Sutton, 2023), meta‐conspiracies do not necessarily challenge mainstream narratives or engage in *denialism* (Uscinski & Klofstad, 2024). However, they still contain an oppositional component for conspiracy believers by suggesting that the *real* conspiracy is the effort to make people believe in conspiracy theories.

Just as real conspiracies gain plausibility through acceptance by official sources, *plausible meta‐conspiracy theories* are distinguished from *implausible* ones for the same reason. Implausible meta‐conspiracies still contradict official explanations for events when alleging that certain conspiracist claims are spread to achieve nefarious goals (e.g. claims that 9/11 was orchestrated by the US government or that the CIA invented the term ‘conspiracy theory’ to silence dissent; Nera et al., 2020). In contrast, comparatively plausible meta‐conspiracies are supported by substantial evidence from credible sources, and thus do not tend to directly challenge official explanations for events. There is strong academic and journalistic consensus that fossil fuel interests have deliberately spread climate denial (Oreskes & Conway, 2011). For instance, the Koch brothers secretly colluded with news media to promote climate change conspiracy theories (Rueles, 2018). Hattersley et al. (2022) further confirmed the perceived relative plausibility of this meta‐conspiracy, showing that participants endorsed it more than other plausible conspiracies, such as Russian secret service poisonings in the UK or Trump's potential collusion with Russia in 2016.

Plausible meta‐conspiracy theories extend beyond climate change. Gorwa (2017) interviewed political campaign managers, journalists and civil society groups, finding that Kremlin‐sponsored computational propaganda in Poland spreads conspiracy theories to weaken institutions and erode trust in truth itself. Similarly, foreign governments have secretly coordinated efforts to spread anti‐vaccination conspiracy theories online for similar purposes (Whiskeyman & Berger, 2021).

## Beliefs and intentions

Inoculation effectively increases resistance to broad conspiracy narratives, such as the oversimplification of complex issues (Mason et al., 2024). We propose that presenting a *plausible meta‐conspiracy theory* can similarly counter specific conspiracy beliefs by appealing to perceptions of a dangerous world where groups secretly collude to spread misinformation (Moulding et al., 2016) and by addressing intergroup antagonism in conspiracist thinking (Biddlestone et al., 2020). Inoculation against conspiracy narratives appears to work regardless of individuals' general conspiracy beliefs (Mason et al., 2024), and interventions are most effective when they provide an alternative explanation to conspiracy narratives (Stasielowicz, 2024). Thus, a *fighting fire with fire* prebunk may not only increase belief in meta‐conspiracy theories among conspiracy‐prone individuals but also reduce conspiracy beliefs by undermining their sources and offering an alternative explanation (Stamatiadis‐Bréhier, 2024).

Evidence suggests that inoculating against specific conspiracy theories may differ in effectiveness from inoculating against general conspiracy narratives – reducing belief in one conspiracy does not always generalize to others (Banas et al., 2023). This distinction is reinforced by recent findings: Inoculation interventions were effective when applied to a specific context (e.g. geothermal energy; Spampatti, Brosch, et al., 2023) but not when targeting climate change misinformation more broadly (Spampatti, Hahnel, et al., 2023). These results underscore the importance of tailoring interventions to concrete narratives rather than abstract themes. Accordingly, while we expect that ‘fighting fire with fire’ will increase beliefs that specific conspiracy theories are fabricated to mislead the public, we remain cautious given the novelty of using meta‐conspiracies as an intervention. However, the implications for behavioural intentions are clearer. Inoculation messages have been shown to increase intentions to vaccinate (Jolley & Douglas, 2017) and support pro‐environmental policies (Spampatti, Brosch, et al., 2023), largely driven by belief shifts induced by persuasive prebunking. Therefore, even if meta‐conspiracy messages promote only weak type‐I suspicions (i.e. that a conspiracy theory might be manipulative) rather than stronger type‐II conclusions (i.e. that a conspiracy theory is definitely false; Dentith, 2022), they may still improve real‐world behavioural intentions in targeted domains like climate action and public health.

We hypothesized that:

*Exposure to specific conspiracy theories would increase belief in specific conspiracy theories compared with the control group*.

*Exposure to prebunking messages suggesting the existence of plausible meta‐conspiracies would increase belief in plausible meta‐conspiracy theories compared with the control group*.

*Exposure to conspiracy theories would reduce relevant behavioural intentions*.

*Exposure to plausible meta‐conspiracy theories would increase relevant behavioural intentions*.

We also examined the effectiveness of *fighting fire with fire* in reducing beliefs in specific conspiracy theories (exploratory in Study 1, preregistered in Study 2) and whether this effect is conditional on general conspiracy belief levels. By testing these hypotheses, we aimed to advance understanding of psychological resistance and acceptance of conspiracy theory interventions. If effective among high conspiracy believers, this approach could enhance prebunking interventions for a group that poses a risk to climate mitigation efforts. If ineffective, it would clarify whether using plausible meta‐conspiracies to engage conspiracist audiences is not a viable strategy.

## OVERVIEW OF THE CURRENT RESEARCH

In this research, we tested an inoculation intervention to increase belief in plausible *meta‐conspiracy theories* about COVID‐19 vaccines (Study 1) and climate change (Study 2). Participants were presented with plausible meta‐conspiracies suggesting that others deliberately spread conspiracy theories on these topics. We call this prebunking technique *fighting fire with fire* and predict that, unlike belief in specific conspiracy theories, belief in these meta‐conspiracies would be positively related to vaccination and pro‐environmental intentions.

In a Pilot Study (see Section 1 in Appendix S1), participants completed a scale measuring belief in plausible COVID‐19 vaccine *meta‐conspiracy theories* (e.g. ‘*Secret groups trying to destabilize society are spreading false information about COVID‐19 vaccines*’), alongside belief in COVID‐19 vaccine conspiracy theories (e.g. ‘*COVID‐19 vaccines are harmful, and this fact is covered up*’) and vaccination intentions. Regression analysis showed that vaccination intentions were negatively related to belief in COVID‐19 vaccine conspiracy theories but positively related to belief in plausible COVID‐19 vaccine meta‐conspiracy theories.

In Study 1, we developed a COVID‐19 vaccine *meta‐conspiracy* intervention to increase belief in plausible meta‐conspiracy theories that challenge the sources and spread of COVID‐19 vaccine conspiracy theories. We tested its effectiveness by presenting the message alone (meta‐conspiracy condition) or alongside a text outlining COVID‐19 vaccine conspiracy theories (before: prebunk, or after it: debunk) and comparing these conditions to a control group and an exposure to COVID‐19 vaccine conspiracy theories condition in a total of five conditions. We also examined whether effects were moderated by the strength of participants' general conspiracy beliefs. In the group that we exposed to COVID‐19 vaccine conspiracy theories, we expected this to increase belief in such theories compared with the control.

In Study 2, we developed a *meta‐conspiracy prebunk* intervention for the climate change context, testing its effects against a standard climate conspiracy prebunk (Cook et al., 2017), exposure to climate conspiracy theories, and an active control condition describing climate scientists' temperature measurement technology in a total of four conditions. We also examined whether general conspiracy beliefs (measured with a single‐item scale; Lantian et al., 2016) and relevant attitudes – such as acceptance of anthropogenic climate change (Lewandowsky et al., 2019) and prescreened climate change beliefs – moderated the intervention's effectiveness.

## STUDY 1

In Study 1, we tested the efficacy of a *plausible COVID‐19 vaccine meta‐conspiracy prebunk*. As preregistered (https://osf.io/za52k/?view_only=6b23edb3913b4df886bb9b05d4fbf45a), we hypothesized that exposure to *only* plausible COVID‐19 vaccine meta‐conspiracy theories would increase both belief in these meta‐conspiracies and vaccination intentions compared with a control group. Conversely, we expected exposure to COVID‐19 vaccine conspiracy theories to increase belief in these conspiracy theories and reduce vaccination intentions. We also explored whether a *prebunk* condition (meta‐conspiracy text before conspiracy text) or a *debunk* condition (conspiracy text before meta‐conspiracy text) would have similar effects to the meta‐conspiracy text alone. Finally, we examined whether the *fighting fire with fire* intervention influenced belief in COVID‐19 vaccine conspiracy theories and whether exposure to COVID‐19 vaccine conspiracy theories affected belief in plausible COVID‐19 vaccine meta‐conspiracies.

In the *only meta‐conspiracy*, *prebunk* and *debunk* conditions, participants were shown variations of a message describing secret groups colluding to spread COVID‐19 vaccine conspiracy theories, presumably to undermine pandemic mitigation efforts in certain societies. Specifically, we presented information from a *Reuters* article detailing an EU investigation, which concluded that the Russian, Chinese and Iranian governments secretly coordinated to spread COVID‐19 vaccine conspiracy theories online in EU nations (Emmott, 2021). We treated this as a plausible example of a covert international conspiracy to disseminate misinformation against political adversaries.

General conspiracy beliefs were taken after the manipulation to assess whether they moderated the intervention's effectiveness. Additionally, we included exploratory predictors based on previous research (e.g. Jolley & Douglas, 2014a, 2014b). These were feelings of powerlessness and uncertainty surrounding COVID‐19.

### Methods

#### Participants

A *priori* power analysis using G*Power estimated that detecting a main effect size similar to Jolley and Douglas's (2014a) study on vaccine conspiracy exposure and vaccination intentions (*d* = 0.37) with *power* = .80 and five conditions required a minimum sample of 585. Due to limitations in specifying participant numbers on Reddit.com, we oversampled, collecting 741 responses (20–21 January 2022) from various US‐related subreddits. Participants were not compensated for their time, and this was clearly outlined in the study information sheet. After excluding non‐US participants (*N* = 12) and those failing attention checks (*N* = 9), the final sample was 720 (*M*_age_ = 37.59; 391 women, 306 men). Conditions included: control (*N* = 152), conspiracy (*N* = 152), prebunk (*N* = 132), debunk (*N* = 143), and meta‐conspiracy (*N* = 141). Sensitivity power analysis (*statsmodels* in Python) indicated that with *N* = 660 across five conditions, using the lowest‐powered condition (*N* = 132, prebunk), a *power* of .90 was achieved to detect a minimum overall main effect of *f* = 0.15 and an experimental post‐hoc effect size of *d* = 0.40 using Tukey and Games‐Howell post‐hoc corrections.

#### Design and procedure

After providing consent, participants were randomly assigned to one of five conditions. In the control condition, participants simply proceeded to the next page without additional reading. In the conspiracy condition, they read a fabricated news excerpt on COVID‐19 vaccine conspiracy theories, adapted from Jolley and Douglas (2014a). This article suggested that people should be suspicious of COVID‐19 vaccines, citing alleged fake vaccination videos, vaccine‐related deaths, and government exaggerations of COVID‐19 risks (see full materials: https://osf.io/xh2fs/?view_only=e31e7c4f5ba4475785364e784f360d53).

In the meta‐conspiracy condition, participants read a fabricated article describing a real case in which Iranian, Russian and Chinese intelligence services were found to have secretly colluded to spread COVID‐19 vaccine conspiracy theories online (Whiskeyman & Berger, 2021). In the prebunk condition, participants first read the meta‐conspiracy text before being exposed to the conspiracy text, allowing us to test whether this sequence enhanced psychological resistance to conspiracy theories (see van der Linden, 2022). In contrast, participants in the debunk condition read the same texts but in reverse order, with the conspiracy text presented before the meta‐conspiracy text.

Next, participants completed the measures assessing their belief in COVID‐19 vaccine conspiracy theories and plausible COVID‐19 vaccine meta‐conspiracy theories, with the order of these scales counterbalanced. They then responded to a measure assessing their vaccination intentions. Following this, participants indicated their general conspiracy beliefs, as well as their feelings of powerlessness and uncertainty regarding COVID‐19, with the order of these measures randomized. Finally, they provided socio‐demographic information and were fully debriefed.

#### Measures

*Belief in COVID‐19 vaccine conspiracy theories* (*M* = 1.50, *SD* = 0.83; *α* = .86) was measured with nine items adapted from Jolley and Douglas (2014a) to the COVID‐19 context (e.g. ‘COVID‐19 vaccines are harmful, and this fact is covered up’), using a response scale from 1 (*strongly disagree*) to 7 (*strongly agree*). Considering the high positive skewness = 2.55 and kurtosis statistics = 7.19, above ±1 for the data distribution of this measure, we implemented bootstrapping (5000 replications) in all subsequent regression analyses.^1^

*Belief in plausible COVID‐19 vaccine meta‐conspiracy theories* (*M* = 4.99, *SD* = 1.33; *α* = .85) was measured with five items capturing elements of the belief that nefarious groups secretly collude to spread false information about COVID‐19 vaccines to achieve their agendas (e.g. ‘Secret groups who are trying to destabilize our society are spreading false information about COVID‐19 vaccines’), using a response scale from 1 (*strongly disagree*) to 7 (*strongly agree*).

*COVID‐19 vaccination intentions* (*M* = 6.04, *SD* = 1.83) were measured with a single item asking participants how likely they would be to accept a hypothetical efficacious vaccine against a new variant of COVID‐19, using a response scale from 1 (*definitely not vaccinate*) to 7 (*definitely vaccinate*).^2^

*General conspiracy beliefs* (*M* = 6.28, *SD* = 1.82; *α* = .80) were measured with the five‐item Conspiracy Mentality Questionnaire (CMQ; Bruder et al., 2013; e.g. ‘[I think that…]…many very important things happen in the world, which the public is never informed about’), using a response scale from 1 (*certainly not*) to 11 (*certain*).

*Powerlessness around COVID‐19* (*M* = 2.92, *SD* = 1.36; *α* = .91) was measured with three items adapted from Jolley and Douglas (2014a; e.g. ‘I feel that the COVID‐19 pandemic is too big for my actions to have an impact’), using a response scale from 1 (*strongly disagree*) to 6 (*strongly agree*).

*Uncertainty around COVID‐19* (*M* = 2.55, *SD* = 1.32; *α* = .68) was measured with three items adapted from Jolley and Douglas (2014a; ‘I feel uncertain about the dangers of COVID‐19’), using a response scale from 1 (*strongly disagree*) to 6 (*strongly agree*).

*Socio‐demographics*. Alongside their age and gender, participants indicated their highest level of completed education (*M* = 5.11; *SD* = 0.67) from 1 (*no formal education*) to 6 (*college or university education (graduate degree)*), religiosity (*M* = 2.07; *SD* = 1.60) using a response scale from 1 (*not at all religious*) to 7 (*very religious*), and their self‐placed levels of general (*M* = 3.33; *SD* = 1.74), social (*M* = 2.83; *SD* = 1.81), and economic conservatism (*M* = 3.87; *SD* = 2.09), with single‐items using response scales from 1 (*extremely liberal*) to 9 (*extremely conservative*). Participants were also asked whether they had any underlying health conditions that may impact the severity of COVID‐19 symptoms (*N* = 220) and whether they were a frontline health care worker (*N* = 45).

#### Ethics

Full ethical approval was obtained through the University of Kent Psychology Research Ethics Committee.

### Results

An exploratory paired samples *t*‐test indicated that plausible COVID‐19 vaccine meta‐conspiracy theories, *M* = 4.99, *SD* = 1.33, were perceived as significantly more plausible than specific COVID‐19 vaccine conspiracy theories, *M* = 1.50, *SD* = 0.82; *t*(719) = 50.44, *p* < .001, *d*_z_ = 1.88.

#### Correlations

Belief in plausible COVID‐19 vaccine meta‐conspiracy theories positively correlated with vaccination intentions and negatively correlated with belief in specific COVID‐19 vaccine conspiracy theories, conspiracy mentality, powerlessness around COVID‐19, and uncertainty around COVID‐19 (see Table 1). In contrast, belief in specific COVID‐19 vaccine conspiracy theories negatively correlated with vaccination intentions and positively correlated with conspiracy mentality, powerlessness around COVID‐19, and uncertainty around COVID‐19 (see Table 1).

**TABLE 1:** Pearson's *r* bivariate correlation coefficients between the main variables in Study 1.

	1	2	3	4	5	6
1. Meta‐conspiracy beliefs	−	−.45***	.50***	−.08*	−.24***	−.31***
2. Conspiracy beliefs		−	−.81***	.50***	.34***	.53***
3. Vaccination intentions			−	−.43***	−.36***	−.53***
4. Conspiracy mentality				−	.29***	.40***
5. COVID‐19 powerlessness					−	.37***
6. COVID‐19 uncertainty						−

*** *p* < .001.

* *p* < .05.

#### Main analyses

##### Conspiracy beliefs

**FIGURE 1:**
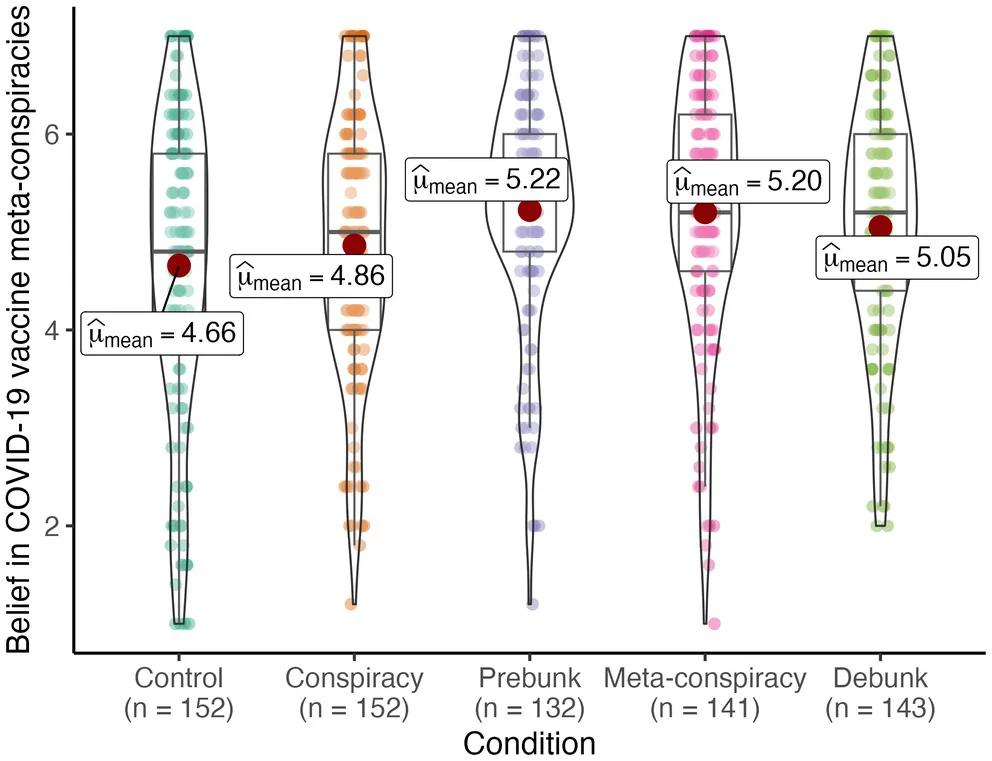
Violin plot of the experimental effect of each condition on belief in plausible COVID‐19 vaccine meta‐conspiracy theories.

To test whether the experimental conditions altered respective conspiracy beliefs, we conducted a series of one‐way ANOVAs with the experimental conditions as the independent variable (five levels) and belief in both COVID‐19 vaccine conspiracy theories and plausible COVID‐19 vaccine meta‐conspiracy theories as respective dependent variables. Levene's tests of homogeneity of variance indicated that this assumption was violated for belief in both COVID‐19 vaccine conspiracy theories, *p* = .010, and plausible COVID‐19 meta‐conspiracy theories, *p* = .011. Therefore, we implemented a Welch test. Results indicated that there were no significant differences between conditions in belief in COVID‐19 vaccine conspiracy theories, *F*(4, 356.89) = 1.34, *p* = .255. However, there was a significant overall main effect of experimental condition on belief in plausible COVID‐19 vaccine meta‐conspiracy theories, *F*(4, 356.58) = 4.30, *p* = .002 (see Figure 1).

Post‐hoc analysis with a Games‐Howell correction for multiple comparisons indicated that belief in plausible COVID‐19 vaccine meta‐conspiracy theories was significantly higher in both the prebunk, *p* = .005, *d* = 0.41, and meta‐conspiracy conditions, *p* = .011, *d* = 0.38, compared with the control group (see Figure 1). The magnitudes of these effect sizes were both small to medium. However, we only had 0.90 power to detect the effect size magnitude of the prebunk (vs. control) effect.

##### Behavioural intentions

Next, we conducted a series of one‐way ANOVAs to investigate the experimental intervention effects on our other hypothesized dependent variables. A Levene's test of homogeneity of variance indicated that this assumption was violated for COVID‐19 vaccination intentions, *p* = .001. Therefore, a Welch test was implemented in the one‐way ANOVA for this variable. There were no significant differences between conditions in COVID‐19 vaccination intentions, *F*(4, 356.60) = 1.70, *p* = .149, or our exploratory dependent variables of uncertainty around COVID‐19, *F*(4, 715) = 0.65, *p* = .626, and powerlessness around COVID‐19, *F*(4, 715) = 0.86, *p* = .490.^3^

##### Exploratory analyses

###### Indirect effects

To explore potential indirect effects of each condition (vs. control) on intentions to accept the COVID‐19 vaccine through beliefs in COVID‐19 vaccine conspiracy theories and plausible meta‐conspiracies, we constructed an indirect path model. In this model, dummy coded variables of each condition (vs. control) were entered as simultaneous predictors, both beliefs in COVID‐19 vaccine conspiracy theories and plausible meta‐conspiracies as simultaneous mediators, and intentions to accept the COVID‐19 vaccine as a dependent variable. We controlled for conspiracy mentality on all paths as well as both powerlessness and uncertainty around COVID‐19 on the mediator paths. There were positive indirect effects of the prebunk (vs. control), standardized indirect effect = .04, 95% CI [0.01, 0.04], and meta‐conspiracy conditions (vs. control), standardized indirect effect = .04, 95% CI [0.01, 0.04], on intentions to accept the COVID‐19 vaccine through beliefs in plausible COVID‐19 vaccine meta‐conspiracies (see Section 2 in Appendix S1). However, there were no significant total effects or other indirect effects on vaccination intentions from the other predictors or through belief in specific COVID‐19 vaccine conspiracy theories (see Section 2 in Appendix S1).

Exploratory regression, moderation, and latent profile analyses can be found in Section 2: Appendix S1. The regression analyses indicated that COVID‐19 vaccination intentions were negatively related to belief in COVID‐19 vaccine conspiracy theories but positively related to belief in plausible COVID‐19 vaccine meta‐conspiracy theories when controlling for the shared variance with general conspiracy beliefs, as well as both uncertainty and powerlessness around COVID‐19. Similarly, the latent profile analysis indicated that those with stronger belief in specific COVID‐19 vaccine conspiracy theories demonstrated lower belief in plausible COVID‐19 vaccine meta‐conspiracy theories. Moderation analyses indicated that there were no significant interaction effects with the prebunk (vs. control) when predicting belief in plausible COVID‐19 vaccine meta‐conspiracy theories.

### Discussion

Study 1 found that *fighting fire with fire* was ineffective in reducing belief in COVID‐19 vaccine conspiracy theories or increasing vaccination intentions. This lack of support for H4 was accompanied by null effects for H1 and H3, as exposure to COVID‐19 vaccine conspiracy theories did not increase belief in these conspiracy theories or reduce vaccination intentions. However, exposure to the *fighting fire with fire* prebunk significantly increased belief in plausible COVID‐19 vaccine meta‐conspiracy theories compared with the control group, supporting H2. Additionally, cross‐sectional regression results mirrored findings from the Pilot Study, showing that vaccination intentions were negatively related to belief in COVID‐19 vaccine conspiracy theories but positively related to belief in plausible meta‐conspiracy theories when controlling for shared variance. Unlike in the Pilot Study, however, beliefs in COVID‐19 vaccine conspiracy theories and plausible meta‐conspiracies were negatively correlated in Study 1.

The findings from Study 1 suggest that shifting beliefs in COVID‐19 vaccine conspiracy theories was particularly difficult in January 2022. This contrasts with two established research areas: one showing that exposure to conspiracy theories increases belief in them (e.g. Jolley et al., 2019; Jolley & Douglas, 2014a, 2014b; Kim & Cao, 2016; van der Linden, 2015) and another demonstrating the effectiveness of prebunking in reducing conspiracy beliefs (e.g. Banas & Miller, 2013; Jolley & Douglas, 2017; Mason et al., 2024). However, Béna et al. (2023) found that repeated exposure to conspiracy theories increases the likelihood of perceiving them as true, and meta‐analytic longitudinal evidence suggests that COVID‐19 vaccine conspiracy beliefs and related behavioural intentions strengthen over time (Stasielowicz, 2022; Bierwiaczonek et al., 2020). Given that our data collection occurred over 2 years into the pandemic, these beliefs may have become particularly entrenched, making them resistant to change in an experimental setting. Additionally, Varet et al. (2024) found that exposure to health‐related conspiracy theories only increased belief when the theories were fictional (Study 2) but not when they reflected real‐world examples (Study 1). This suggests a potential disconnect in experimental research, as isolating the effects of real‐world conspiracy theories may be difficult once they have already spread into public consciousness.

While *fighting fire with fire* did not reduce belief in COVID‐19 vaccine conspiracy theories, it also did not increase belief in them, suggesting it carries no risk of backfiring. This may indicate that the intervention induces weak *type‐I* suspicion – raising doubts about the origins and spread of vaccine conspiracy theories – without fully discrediting them through stronger *type‐II* suspicion (Dentith, 2022). However, given the accompanying null effects, this remains uncertain. Despite this, increasing belief in plausible meta‐conspiracy theories that question the origins of conspiracy theories and positively relate to behavioural intentions is unlikely to increase susceptibility to those same conspiracy theories. Whether this applies to unrelated conspiracy theories remains unclear. Exploratory moderation analyses also showed that the *fighting fire with fire* prebunk significantly increased belief in plausible COVID‐19 vaccine meta‐conspiracy theories compared with the control condition, regardless of participants' prior general conspiracy beliefs (see Section 2 in Appendix S1). This suggests that the intervention works independently of baseline conspiracy susceptibility.

To extend these findings to a topic more relevant to this special issue, we next examined the effectiveness of *fighting fire with fire* in the context of climate change conspiracy theories.

## STUDY 2

Unlike previous research (e.g. Bierwiaczonek et al., 2022; van der Linden, 2015; cf. de Saint Laurent et al., 2022), Study 1 did not find the expected effects of exposure to COVID‐19 vaccine conspiracy theories on conspiracy beliefs or vaccination intentions. This may be due to the increasing entrenchment of COVID‐19 conspiracy beliefs by 2022, making them harder to shift compared with their initial spread. This raises concerns about the limitations of experimental designs in studying temporally relevant conspiracy theories (Bierwiaczonek et al., 2022; Stasielowicz, 2022; Verat et al., 2023). To address this, Study 2 shifted focus to the *fighting fire with fire* prebunk intervention in the climate change context, where conspiracy beliefs are likely to be more temporally stable.

In Study 1, we were unable to compare the effects of *fighting fire with fire* against standard prebunking messages commonly used in the literature. To address this, Study 2 included a standard prebunk condition alongside the *fighting fire with fire* prebunk in a four‐cell design, allowing us to examine whether introducing a meta‐conspiracy framing produces different effects compared with standard prebunks that simply allude to manipulative groups or individuals. We also modified several aspects of the study design. The measure of general conspiracy beliefs was replaced with a single‐item scale (Lantian et al., 2016) to reduce completion time. Additionally, Study 1's control condition was passive, meaning we could not determine what participants had in mind while completing the survey. To improve this, Study 2 included an *active* control condition in which participants read a text about climate science technology used to measure historical Earth temperatures. Finally, to obtain a more representative sample and mitigate the ‘access problem’ (Wood & Douglas, 2015), we prescreened participants using *Prolific*, ensuring that half identified as believing in climate change and half as not believing in it.^4^

In line with our preregistration (https://osf.io/ruq8g/?view_only=b9487ae3fe51470dadd63fcb2af30a35), we hypothesized that a *fighting fire with fire* climate change prebunk would significantly increase belief in plausible climate change meta‐conspiracy theories and decrease belief in climate change conspiracy theories compared with the control group. We also expected that a standard prebunk would reduce belief in climate change conspiracy theories and increase perceived scientific consensus on anthropogenic climate change relative to the control group. Additionally, we hypothesized that exposure to climate change conspiracy theories would increase belief in these conspiracy theories while decreasing both intentions to reduce one's carbon footprint and perceived scientific consensus on anthropogenic climate change. Finally, we explored whether these experimental effects were moderated by general conspiracy beliefs, prescreened climate change acceptance versus denial, or belief in anthropogenic climate change.

### Methods

#### Participants

A *priori* power analysis estimated that detecting a knockout interaction effect, using an effect size comparable with the relationship between belief in plausible COVID‐19 vaccine meta‐conspiracy theories and vaccination intentions from Study 1 (*r* = .17) with *power* = .80 and four conditions, a minimum sample of 1076 participants was required. A total of 1145 US *Prolific* workers participated. After removing duplicates (*N* = 13), incomplete responses (*N* = 66) and those failing attention checks (*N* = 36), the final sample included 1077 participants (*N* = 540 climate accepters, *N* = 75 climate skeptics, *N* = 462 climate deniers; 531 men, 539 women; *M*_age_ = 45.88). A sensitivity power analysis (*statsmodels* in Python) indicated that with a total sample of 1028 across four conditions, using the lowest‐powered condition (*N* = 257, standard prebunk), *power* = .90 was achieved to detect a minimum overall main effect of *f* = 0.12 and an experimental post‐hoc effect size of *d* = 0.14 between each condition and the control using Tukey or Games‐Howell post‐hoc corrections.

#### Design and procedure

After providing consent, participants were randomly assigned to one of four conditions. In the control condition, they read an excerpt from a UK Research and Innovation (*UKRI*)‐funded article about the technology used to measure historical Earth temperatures (*full materials*: https://osf.io/xh2fs/?view_only=e31e7c4f5ba4475785364e784f360d53). In the conspiracy condition, participants read a fabricated article adapted from Jolley and Douglas (2014b) suggesting that climate scientists have financial incentives to lie about their findings to secure research funding. In the *standard prebunk* condition, they first read a text adapted from Cook et al. (2017) explaining how fossil fuel industries and political groups spread misinformation to create false scientific debate and delay climate action. They then read the same conspiracy text as in the conspiracy condition to test their psychological immunity against climate change conspiracy theories. In the *fighting fire with fire* prebunk condition, participants read a fabricated article inspired by real Greenpeace investigations (see Greenpeace, 2010), describing how the Koch brothers – owners of a major fossil fuel conglomerate – secretly colluded with media companies to spread climate conspiracy theories. To reinforce the conspiratorial framing, the text included phrases such as ‘*Remember to question whether the real conspiracy comes from those promoting conspiracy theories*’ and the classic trope ‘*Follow the money!*’ (see Stamatiadis‐Bréhier, 2024). As in the standard prebunk condition, they then read the conspiracy text to assess their psychological resistance to climate conspiracy theories.^5^

Next, participants completed measures assessing their belief in climate change conspiracy theories, belief in plausible climate change meta‐conspiracy theories, and perceived scientific consensus on anthropogenic climate change, with the order of these scales randomized. They then responded to a measure assessing their intentions to reduce their carbon footprint.^6^ Finally, participants were presented with text explaining they were moving on to the final part of the study before reporting their general conspiracy beliefs, existing beliefs about anthropogenic climate change, and their socio‐demographic information. They were fully debriefed at the end of the study.

#### Measures

*Belief in climate change conspiracy theories* (*M* = 3.75, *SD* = 2.07; *α* = .98) was measured with Jolley and Douglas' (2014b) seven‐item belief in climate change conspiracy theories scale (e.g. ‘Climate change is a hoax’), using a response scale from 1 (*strongly disagree*) to 7 (*strongly agree*). Once again, considering the high negative kurtosis statistic = −1.41, above ±1 for the data distribution of this variable (but normal skewness statistic = 0.04), we implemented bootstrapping (5000 replications) in all subsequent regression analyses.

*Belief in plausible climate change meta‐conspiracy theories* (*M* = 3.99, *SD* = 1.59; *α* = .90) was measured by adapting the five‐item belief in plausible COVID‐19 vaccine meta‐conspiracy theories scale from Study 1 to the climate change context (e.g. ‘Oil and gas companies spread climate change conspiracy theories to cast doubt on human‐made climate change and boost their profits’), using a response scale from 1 (*strongly disagree*) to 7 (*strongly agree*).

*Perceived scientific consensus on anthropogenic climate change* (*M* = 67.00, *SD* = 25.13) was measured by asking participants to estimate the percentage of climate scientists who agree that climate change is caused by human activity (e.g. burning fossil fuels) using a single sliding scale from 0 to 100.

*Intentions to reduce one*'*s carbon footprint* (*M* = 3.45, *SD* = 1.60; *α* = .88) were measured with Jolley and Douglas' (2014b) scale asking participants the extent to which they intended to take part in seven pro‐environmental behaviours over the next 12 months (e.g. ‘Plant a tree’) using a response scale from 1 (*definitely not*) to 7 (*definitely yes*).

*General conspiracy beliefs* (*M* = 5.70, *SD* = 2.48) were measured using Lantian et al.'s (2016) single‐item conspiracy beliefs scale (SICBS), which first presents a preamble priming conspiracy theories:

Some political and social events are debated (for example 9/11 attacks, the death of Lady Diana, the assassination of John F. Kennedy). It is suggested that the ‘official version’ of these events could be an attempt to hide the truth from the public. This ‘official version’ could mask the fact that these events have been planned and secretly prepared by a covert alliance of powerful individuals or organizations (for example secret services or government). What do you think?

Next, participants are asked to indicate their agreement with the statement ‘I think that the official version of the events given by the authorities very often hides the truth’ using a response scale from 1 (*completely false*) to 9 (*completely true*).

*Belief in anthropogenic climate change* (*M* = 4.11, *SD* = 1.85; *α* = .93) was measured with Lewandowsky et al.'s (2019) five‐item scale (e.g. ‘Human CO2 emissions cause climate change’) using a response scale from 1 (*strongly disagree*) to 7 (*strongly agree*).

*Socio‐demographics* were measured as in Study 1, capturing participants' highest levels of completed education (*M* = 4.80, *SD* = 0.73), religiosity (*M* = 3.56, *SD* = 2.25) and their self‐placed general (*M* = 5.36, *SD* = 2.56), social (*M* = 5.16, *SD* = 2.67), and economic conservatism (*M* = 5.66, *SD* = 2.61).

#### Ethics

Full ethical approval was obtained through the University of Kent Psychology Research Ethics Committee.

### Results

An exploratory paired‐samples *t*‐test indicated that plausible climate change meta‐conspiracy theories, *M* = 3.99, *SD* = 1.59, were perceived as significantly more plausible than specific climate change conspiracy theories, *M* = 3.75, *SD* = 2.07; *t*(1075) = 2.58, *p* = .010, *d*_z_ = 0.08.^7^

#### Prescreen checks

A series of one‐way ANOVAs with the prescreen categories as the independent variable (−1 = climate change deniers, 0 = climate change skeptics, 1 = climate change accepters) indicated that there were significant differences between these groups' general conspiracy beliefs, *F*(2, 1074) = 71.77, *p* < .001, and belief in anthropogenic climate change, *F*(2, 1074) = 852.99, *p* < .001 (see Appendix S1: Figures S14 and S15). Post‐hoc analyses with Tukey corrections for multiple comparisons indicated that climate deniers had significantly stronger general conspiracy beliefs than both climate sceptics and accepters, but there was no significant difference between climate sceptics and accepters. They also believed less in anthropogenic climate change than both climate sceptics and accepters (see Appendix S1: Figures S14 and S15).

#### Correlations

Belief in plausible climate change meta‐conspiracy theories negatively correlated with belief in specific climate change conspiracy theories, and positively correlated with climate intentions, perceived scientific consensus around climate change, and acceptance of anthropogenic climate change (see Table 2). The correlation between belief in plausible climate change meta‐conspiracy theories and general conspiracy beliefs was non‐significant (see Table 2). In contrast, belief in specific climate change conspiracy theories positively correlated with general conspiracy beliefs, and negatively correlated with climate intentions, perceived scientific consensus around climate change, and acceptance of anthropogenic climate change (see Table 2).

**TABLE 2:** Pearson's *r* bivariate correlation coefficients between the main variables in Study 2.

	1	2	3	4	5	6
1. Meta‐conspiracy beliefs	–	−.37***	.37***	−.03	.22***	.48***
2. Conspiracy beliefs		–	−.53***	.47***	−.47***	−.89***
3. Climate intentions			–	−.18***	.24***	.58***
4. General conspiracy beliefs				–	−.24***	−.41***
5. Perceived scientific consensus					–	.50***
6. Acceptance of anthropogenic climate change						–

*** *p* < .001.

#### Main analyses

##### Conspiracy beliefs

**FIGURE 2:**
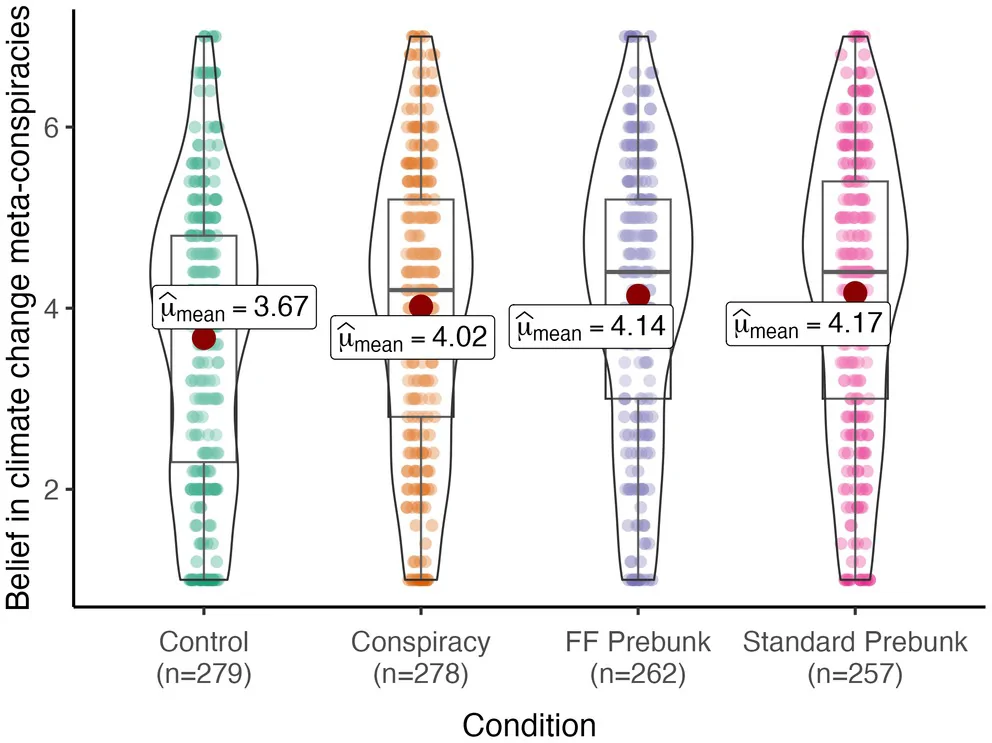
Violin plot of the experimental effect of each condition on climate change meta‐conspiracy beliefs (Study 2). FF prebunk, fighting fire with fire prebunk.

Levene's tests of homogeneity of variance indicated that this assumption was violated for belief in both climate change conspiracy theories, *p* = .011, and plausible climate change meta‐conspiracy theories, *p* = .031. Therefore, Welch tests were implemented in their analyses. A one‐way ANOVA with the experimental conditions as the independent variable indicated that there were no significant differences between conditions in belief in climate change conspiracy theories, *F*(3, 594.69) = 1.18, *p* = .316. However, there were significant differences between conditions in belief in plausible climate change meta‐conspiracy theories, *F*(3, 594.11) = 5.63, *p* < .001. Post‐hoc analysis with a Games‐Howell correction for multiple comparisons indicated that beliefs in plausible climate change meta‐conspiracy theories were significantly higher in the fighting fire with fire prebunk, *p* = .004, *d* = 0.30, the standard prebunk, *p* = .002, *d* = 0.31, and the conspiracy conditions, *p* = .048, *d* = 0.22, compared with the control group (see Figure 2).

##### Climate intentions

Levene's tests of homogeneity of variance indicated that this assumption was violated for perceived scientific consensus on anthropogenic climate change, *p* < .001. Therefore, a Welch test was implemented for this analysis. There were no significant differences between conditions in perceived scientific consensus on anthropogenic climate change, *F*(3, 590.99) = 0.48, *p* = .694, or intentions to reduce one's carbon footprint, *F*(3, 1073) = 0.82, *p* = .484.

#### Exploratory analyses

Indirect path models controlling for perceived scientific consensus on the mediator paths indicated that the fighting fire with fire prebunk (vs. control), standardized indirect effect = .04, 95% CI [0.01, 0.07], standard prebunk (vs. control), standardized indirect effect = .06, 95% CI [0.02, 0.010] and exposure to climate change conspiracy theories (vs. control) condition, standardized indirect effect = .03, 95% CI [0.01, 0.05], had positive indirect effects on climate intentions through belief in plausible climate change meta‐conspiracy theories (see Section 3 in Appendix S1).

Regression analysis indicated similar findings to the Pilot Study and Study 1, wherein climate intentions were negatively predicted by belief in climate change conspiracy theories, β = −0.13, *B* = −0.10, *SE* = 0.04, 95% CI [−0.19, −0.02], and positively predicted by belief in plausible climate change meta‐conspiracy theories, *β* = 0.11, *B* = 0.11, *SE* = 0.03, 95% CI [0.05, 0.17], when controlling for their shared variance with general conspiracy beliefs, perceived scientific consensus on anthropogenic climate change, and belief in anthropogenic climate change (see also Appendix S1: Figure S22, for latent profile analysis).

##### Interaction effects

To explore whether the significant experimental effects of condition (fighting fire with fire prebunk vs. control, standard prebunk vs. control, and conspiracy vs. control) on belief in plausible climate change meta‐conspiracy theories were conditional on prior beliefs (general conspiracy beliefs, prescreened climate acceptance, belief in anthropogenic climate change) or socio‐demographics, we conducted moderation analyses with these variables as moderators. For brevity, only summaries of the main moderation analyses are presented here; details of the moderation analyses can be found in Figures S16–S21 of Appendix S1. The moderation analyses indicated that the positive effect of the standard prebunk on belief in climate change meta‐conspiracies was only significant at low and moderate levels of general conspiracy beliefs, moderate and higher levels of belief in anthropogenic climate change, and only among climate accepters and skeptics, but not deniers. In contrast, the positive effect of the fighting fire with fire condition on belief in climate change meta‐conspiracies was significant at all levels of general conspiracy beliefs, belief in anthropogenic climate change, or prescreened climate acceptance (see Section 3 in Appendix S1).

### Discussion

Once again, the findings from Study 2 suggest that *fighting fire with fire* is not an effective strategy for reducing conspiracy beliefs, this time in the climate change context. The *fighting fire with fire* prebunk had no effect on belief in climate change conspiracy theories or intentions to reduce one's carbon footprint. Additionally, exposure to climate change conspiracy theories did not increase belief in these conspiracy theories or reduce climate intentions. However, the conspiracy condition, as well as both prebunking interventions, significantly increased belief in plausible climate change meta‐conspiracy theories relative to the control group. Consistent with the Pilot Study and Study 1, climate intentions were negatively related to belief in climate change conspiracy theories but positively related to belief in plausible meta‐conspiracy theories. As in Study 1 – but not the Pilot Study – belief in conspiracy theories was negatively correlated with belief in plausible meta‐conspiracy theories. Moderation analyses revealed that the *fighting fire with fire* prebunk increased belief in plausible meta‐conspiracies regardless of participants' prior general conspiracy beliefs or climate perceptions. In contrast, the standard prebunk only increased these beliefs among those with low or moderate general conspiracy beliefs or those who already accepted anthropogenic climate change to a moderate or strong degree.

Once again, in contrast to a well‐established body of research (e.g. Biddlestone et al., 2022; Jolley & Douglas, 2014b; van der Linden, 2015), exposure to climate change conspiracy theories did not alter participants' climate conspiracy beliefs or intentions. Notably, much of this prior evidence was collected before 2020. More recent findings align more closely with our results. Bolsen et al. (2019) found that exposure to climate conspiracy rhetoric did not influence beliefs in Study 1 and only increased them among Democrat voters in Study 2 (see also Varet et al., 2024). Given the strong partisan and ideological links between conservatism and climate conspiracy beliefs (Biddlestone & Azevedo, 2024; Enders et al., 2022), it is possible that the increasing politicization of climate change conspiracy theories has strengthened ideological resistance to shifting these beliefs in recent years. Notably, neither exposure to climate conspiracy theories nor the *fighting fire with fire* prebunk altered climate conspiracy beliefs, but neither did the standard prebunk – despite its prior effectiveness in studies conducted before 2020 (Cook et al., 2017). Furthermore, the *fighting fire with fire* prebunk did not increase belief in plausible climate change meta‐conspiracy theories among more economically conservative participants (see Figure S16 in Appendix S1), reinforcing the idea that ideological resistance plays a role.

Despite the null effects on climate change conspiracy beliefs, moderation analyses suggest that *fighting fire with fire* has a unique influence not captured by standard prebunking messages. While the standard prebunk did not increase belief in plausible climate change meta‐conspiracy theories among those low in general conspiracy beliefs or those who rejected anthropogenic climate change, *fighting fire with fire* increased meta‐conspiracy beliefs regardless of these prior perceptions. This suggests that the conspiracist framing used in *fighting fire with fire* was at least qualitatively more conspiratorial than the standard prebunk and, at best, more persuasive among target populations when introducing novel alternative conspiracy explanations (Stasielowicz, 2024). Additionally, our cross‐sectional findings confirmed a negative relationship between belief in plausible meta‐conspiracy theories and climate conspiracy beliefs. This indicates that exposure to plausible meta‐conspiracies does not appear to increase general conspiracy suspicions, likely because these meta‐conspiracies challenge the legitimacy of existing conspiracy theories.

## GENERAL DISCUSSION

In this research, we developed a novel prebunking approach called *fighting fire with fire*, introducing a plausible meta‐conspiracy theory suggesting that secret groups deliberately spread conspiracy theories for their own agendas. In the cross‐sectional pilot study, belief in COVID‐19 vaccine conspiracy theories correlated positively with belief in plausible vaccine meta‐conspiracy theories. However, when controlling for shared variance, belief in vaccine conspiracy theories was negatively related to vaccination intentions, while belief in plausible meta‐conspiracy theories was positively related. In Studies 1 and 2, the *fighting fire with fire* prebunk did not reduce belief in COVID‐19 vaccine (Study 1) or climate change (Study 2) conspiracy theories, only increasing belief in plausible meta‐conspiracy theories compared with the control group. Exploratory indirect path models demonstrated that the intervention indirectly increased intentions to vaccinate (Study 1) and reduce one's carbon footprint (Study 2) through beliefs in plausible meta‐conspiracy theories. However, these findings should be treated with caution as causation cannot be inferred between the mediator variables and outcome variables.

The positive effect of fighting fire with fire on belief in plausible meta‐conspiracy theories was observed regardless of general conspiracy beliefs (Studies 1 and 2), belief in anthropogenic climate change (Study 2), or prescreened climate change acceptance or rejection (Study 2). In contrast, the standard prebunk in Study 2 only increased belief in plausible meta‐conspiracy theories among those with weaker or more moderate conspiracy beliefs and stronger or more moderate climate change acceptance. It had no effect on strong conspiracy believers, weaker climate change believers, or climate change deniers. Although previous research suggests conspiracy exposure or interventions can alter conspiracy beliefs (e.g. Jolley & Douglas, 2014a, 2014b), our findings indicate that *fighting fire with fire* may engage participants with strong prior suspicions differently. We argue that those who have stronger prior conspiracy beliefs may find meta‐conspiracy narratives more appealing than those with lower prior conspiracy suspicions due to their comparable narrative structures (i.e. uncovering hidden plots) and their resulting appeal to satisfy the same unmet psychological needs as specific conspiracy theories (see Douglas et al., 2017, 2019). Since belief in plausible meta‐conspiracy theories correlated positively with relevant behavioural intentions and negatively with specific conspiracy beliefs, these findings could inform future interventions.

The standard prebunk in Study 2 neither reduced belief in specific conspiracy theories nor increased belief in plausible climate change meta‐conspiracies among strong conspiracy believers. However, similar prebunking messages have previously been shown to improve resistance to conspiracy narratives regardless of prior attitudes (e.g. Mason et al., 2024). This discrepancy may stem from our effort to circumvent the *access problem* (Wood & Douglas, 2015), where conspiracy believers' distrust of scientific researchers makes them underrepresented in studies. To address this, we prescreened participants so that half were climate accepters and the other half were climate skeptics or deniers. As expected, climate deniers exhibited significantly stronger general conspiracy beliefs and lower climate change acceptance. Thus, null effects may be more common in studies with similarly diverse samples.

The *fighting fire with fire* prebunk increased belief in plausible meta‐conspiracy theories but did not alter belief in conspiracy theories. This may be due to entrenched COVID‐19 conspiracy beliefs (Stasielowicz, 2022) or growing ideological resistance to changing climate conspiracy beliefs (Bolsen et al., 2019; van der Linden et al., 2017). Additionally, Varet et al. (2024) found that exposure to health‐related conspiracy theories only increased conspiracy beliefs when the theories were fictional but not when they reflected real‐world content. This suggests that had Mason et al. (2024) examined specific conspiracy theories rather than general conspiracist narratives, they may have found it more difficult to reduce these beliefs.

### Limitations and future directions

Despite the effectiveness of *fighting fire with fire* in increasing belief in plausible meta‐conspiracies – oppositional to specific conspiracy beliefs – important ethical questions remain. Is it responsible to encourage conspiracist suspicions, even if constructively? Our intervention only increased belief in meta‐conspiracies supported by substantial evidence (e.g. Greenpeace, 2010; Whiskeyman & Berger, 2021), but concerns about legitimizing conspiracy theories more generally may remain. We argue that ignoring real conspiracies risks alienating the populations researchers need to engage with to counter misinformation. Similar to findings on communicating evidential uncertainty to maintain trust in information sources (Batteux et al., 2022; Dries et al., 2024), acknowledging real conspiracies may foster better dialogue with conspiracy believers. However, this approach may itself be seen as a conspiracy to spread meta‐conspiracy theories, though it could still encourage caution when people evaluate conspiracist claims in the future.

The pilot study found a positive correlation between belief in vaccine conspiracy theories and plausible meta‐conspiracy theories, but in the intervention studies, this correlation was consistently negative, suggesting that *fighting fire with fire* does not reduce conspiracy beliefs but also does not increase them. This aligns with Dentith's (2022) concept of *weak type‐I suspicions*, which introduce doubt without fully discrediting a belief. Another consideration is that participants had likely already encountered the conspiracy narratives that were prebunked in Studies 1 and 2. While research suggests that both prophylactic (i.e. before the misinformation has been encountered in one's life) and therapeutic (i.e. after the misinformation has been encountered in one's life) prebunking can be effective (e.g. Compton, 2020), therapeutic prebunking may be limited to generic or technique‐based messaging (see Amazeen et al., 2022). Therefore, future research would benefit from comparing the effectiveness of *fighting fire with fire* with both prophylactic and therapeutic prebunking messages to better understand its parameters.

One notable finding is that while the *fighting fire with fire* intervention did not directly increase behavioural intentions (e.g. pro‐environmental action), indirect path analyses revealed that intentions were positively predicted by increased belief in plausible meta‐conspiracy theories in both studies, but not by changes in belief in specific conspiracy theories. This suggests a meaningful indirect route through which the intervention may shape behaviour, even if those effects are not immediately observable at the level of direct influence. However, the absence of a direct effect may reflect the motivational or conceptual distance between the intervention content (i.e. recognizing that powerful actors spread conspiracy theories) and the specific behavioural outcomes we measured (e.g. planting a tree). Furthermore, we cannot assume causation between the mediator variables and outcome variables in these indirect path analyses. Acknowledging systemic manipulation may not necessarily translate into individual‐level action, particularly when behaviours are effortful or not tightly framed as responses to that manipulation (see Nisa et al., 2019). This highlights the need for future iterations of the intervention to include stronger behavioural framing or action cues, and to closely align belief‐change content with concrete behavioural recommendations, thereby closing the gap between belief and action (see Göral & Hannum, 2024).

Belief in plausible climate change meta‐conspiracy theories also increased after exposure to climate change conspiracy theories in Study 2. This may be due to the higher perceived plausibility of meta‐conspiracy theories compared with specific conspiracy theories indicated in both intervention studies (see also Hattersley et al., 2022). Interestingly, when compared to the COVID‐19 context (Study 1), specific climate change conspiracy beliefs sat higher overall in Study 2 – likely reflecting the chronic, politicized nature of climate attitudes and our prescreen that – unlike Study 1 – actively included sceptics and deniers. As a result, although endorsement of climate meta‐conspiracy items still scored around the midpoint and (on average) higher than belief in specific climate conspiracy theories, the between‐construct gap was smaller. This pattern is consistent with entrenched domain beliefs and motivated‐reasoning dynamics among climate sceptics that sustain comparatively elevated endorsement of specific climate conspiracy claims.

While people may hold contradictory conspiracy beliefs (Miani et al., 2024; Wood et al., 2012; but see van Prooijen et al., 2023 for evidence that claims of belief in contradictory conspiracy theories may be exaggerated), emphasizing plausible meta‐conspiracies may selectively increase their appeal without amplifying specific conspiracy theories. This is likely because meta‐conspiracies challenge the origins of specific conspiracies, but not vice versa (Stamatiadis‐Bréhier, 2024). Supporting this, Hattersley et al. (2022) found plausible conspiracy beliefs cannot be collapsed onto broader conspiracy beliefs, suggesting distinct psychological mechanisms.

Another limitation is that we did not collect measures of participants' general conspiracy beliefs prior to the intervention. We deliberately placed these items after the manipulation, with a break including information intended to semantically separate the intervention section from these variables for participants to avoid priming effects that could contaminate responses to the main outcome measures. This design choice means we cannot strictly rule out preintervention between‐group differences in general conspiracy beliefs. However, we found no significant differences in general conspiracy beliefs between conditions when measured post‐intervention in both intervention studies, suggesting that the intervention did not contaminate those beliefs and that these measures likely reflect participants' prior general conspiracy beliefs. Future work could benefit from incorporating both pre‐ and post‐intervention assessments to more firmly establish baseline equivalence and potential change.

Another key consideration is the *source* of the *fighting fire with fire* intervention. In both prebunking messages, we omitted references to the institutions that uncovered these conspiracies – the EU (Study 1) and Greenpeace (Study 2) – to minimize confounding effects related to participants' perceptions. However, in real‐world settings, removing source attribution is unrealistic. Stamatiadis‐Bréhier (2024) argues that the credibility of sources behind *second‐order conspiracy theories* is central to their persuasive power. Conspiracy believers rarely uncover real conspiracies themselves (Veisdal, 2022), which are instead revealed by investigative journalists – figures often dismissed as part of the ‘mainstream media’. Yet, presenting a meta‐conspiracy as deliberate misinformation could still be perceived as oppositional. Stasielowicz (2024) notes that such interventions challenge individuals' epistemic realities, potentially reinforcing resistance rather than reducing conspiracy beliefs. The applicability of *fighting fire with fire* in contexts other than the US may also produce varied effects.

Other epistemic concerns may be raised regarding the influence of information architecture on the effectiveness of *fighting fire with fire*. Pierre's (2020) *two‐component socio‐epistemic model* suggests *epistemic mistrust* is central to conspiracy belief. Once individuals reject official sources, an *epistemic vacuum* makes them more susceptible to biased information. Thus, the real‐world effectiveness of *fighting fire with fire* may depend on framing the intervention as coming from an alternative *trusted* source. Future research could explore source effects (e.g. mainstream vs. alternative media) and test the intervention beyond the United States to assess its broader applicability (Traberg & van der Linden, 2022). Finally, while fighting fire with fire may address the personal conspiracy beliefs of participants with the implementation of psychological inoculation, other work suggests that addressing unmet psychological needs may be another vital focus for interventions (e.g. Biddlestone et al., 2025). Therefore, we recommend that future work on psychological inoculation examines the possibility of addressing psychological needs, such as those related to achieving epistemic certainty (e.g. Biddlestone, Roozenbeek, & van der Linden, 2022; Vraga et al., 2019).

## CONCLUSION

We introduce *fighting fire with fire*, a novel prebunking approach that increases belief in plausible meta‐conspiracy theories questioning the origins of specific conspiracy theories. Unlike the standard prebunk, fighting fire with fire increased belief in plausible meta‐conspiracies regardless of prior general conspiracy beliefs. However, careful development is needed to ensure only well‐evidenced meta‐conspiracies are used, preventing the replacement of one conspiracy belief with another. We hope this approach advances prebunking strategies, particularly for global crises like climate change, by enhancing message appeal among key populations.

## AUTHOR CONTRIBUTIONS

**Mikey Biddlestone:** Conceptualization; investigation; writing – original draft; methodology; validation; visualization; writing – review and editing; software; formal analysis; data curation. **Ricky Green:** Conceptualization; investigation; writing – review and editing. **Daniel Toribio‐Flórez:** Writing – review and editing. **Dylan de Gourville:** Writing – review and editing. **Robbie M. Sutton:** Conceptualization; investigation; writing – review and editing; methodology; supervision. **Karen M. Douglas:** Supervision; writing – review and editing; conceptualization; methodology; funding acquisition; investigation.

## FUNDING INFORMATION

This research was supported by the European Research Council Advanced Grant Council of conspiracy theories – CONSPIRACY_FX. Number: 101018262, awarded to the last author.

## Supporting information


Appendix S1.

## Data Availability

The data that support the findings of this study are openly available in OSF at: https://osf.io/xh2fs/?view_only=48e49b86ffb644a6b102992724bfc3ed.

## Footnotes

^1^
An attention check item instructed participants to select the seventh option, *‘strongly agree’*, to confirm attentiveness. Those who failed to do so were excluded from analysis.

^2^
Before being presented with this measure, participants were asked ‘Have you been vaccinated against COVID‐19?’ They selected their response from the options ‘No, I have not been vaccinated at all’. (*N* = 52), ‘Yes, I have had two doses and a third dose or booster’ (*N* = 6), ‘Yes, but I have not had my second dose and I do not intend to have it’ (*N* = 2), ‘Yes, but I have not had my second dose and I do intend to have it’ (*N* = 33), ‘Yes, but I have not had my third dose or booster and I do intend to have it’ (*N* = 564), ‘Yes, but I have not had my third dose or booster and I do not intend to have it’ (*N* = 0). This measure was intended to be included as a covariate, but the disparity in sample size differences between responses rendered this variable unusable.

^3^
A robustness check also confirmed no significant differences in general conspiracy beliefs between conditions, *F*(4, 715) = 1.57, *p* = .179.

^4^
Although our pre‐registration specified two groups (climate deniers and climate accepters), fully recruiting climate deniers on *Prolific* proved challenging. To address this, we supplemented the sample with a small number of climate sceptics (those who selected *‘do not know’* in the pre‐screening surveys).

^5^
After reading the texts, participants answered an attention check question to recall key information. For the control text, they identified how scientists measure past surface temperatures (*answer: ‘…by analyzing the ratio of “heavy” atoms of oxygen and hydrogen’*). For the conspiracy text, they recalled why scientists might manipulate climate data (*answer: ‘…to keep their research funding program going’*). For the *fighting fire with fire* prebunk, they identified who secretly colluded with news companies to spread false climate claims (*answer: ‘…the Koch brothers’*). For the standard prebunk, they recalled who pioneered strategies to cast doubt on human‐caused climate change (*answer: ‘…the tobacco industry in the 1970s’*). Participants who failed these checks were excluded from analysis.

^6^
Participants also completed items measuring attitudinal ambivalence toward climate change policies for a separate project (*survey materials*: https://osf.io/xh2fs/?view_only=e31e7c4f5ba4475785364e784f360d53).

^7^
A robustness check also confirmed no significant differences in general conspiracy beliefs between conditions, *F*(3, 1073) = 1.41, *p* = .239.
