# Supplementary material for: Fighting fire with fire: Prebunking with the use of a plausible meta‐conspiracy framing
Source: Br J Psychol. 2025 Sep 8;117(Suppl 1):S113–36. doi: 10.1111/bjop.70023 (PMC13543330; doi:10.1111/bjop.70023)
Supplement: Supplementary file 1 [file BJOP-117-S113-s001.docx]

**Fighting fire with fire: The use of conspiracy framing to improve the efficacy of conspiracy theory pre-bunks**

**Supplementary Materials**

**Section 1: Pilot Study**

We aimed to explore the relationships between beliefs in plausible COVID-19 vaccine meta-conspiracy theories, specific COVID-19 vaccine conspiracy theories, and intentions to accept the COVID-19 vaccine. While this study was exploratory, and thus we did not pre-register our hypotheses, we expected that belief in specific COVID-19 vaccine conspiracy theories would be positively related to belief in plausible COVID-19 vaccine meta-conspiracy theories since they both represent susceptibility to conspiracy narratives. However, when entered into a regression model as simultaneous predictors, we expected that intentions to accept the COVID-19 vaccine would be negatively related to belief in COVID-19 vaccine conspiracy theories (due to their narratives undermining the efficacy and safety of the vaccines), whereas belief in plausible COVID-19 vaccine meta-conspiracy theories would be positively related to intentions to accept the COVID-19 vaccine (due to the implication that conspiracy theories regarding COVID-19 vaccines are simply forms of disinformation secretly being spread to serve the nefarious agendas of secret groups).

**Methods**

***Participants***

A total of 480 responses were collected for the purposes of a wider survey from *Amazon MTurk* on 5^th^ May 2021. Once participants were removed for not reporting their age, reporting being under the age of 18, or indicating that they were not from the US, the final sample consisted of 438 participants, 161 women, 276 men, *M_age_* = 37.01. Sensitivity power analysis with G*Power indicated that this sample size achieved a power of .90 to detect a minimum correlational effect size of *r* = .15.

***Ethics***

Full ethical approval was obtained through BLINDED.

***Design and procedure***

After providing their consent, participants completed the measures of belief in COVID-19 vaccine conspiracy theories and belief in plausible COVID-19 vaccine meta-conspiracy theories (counterbalanced). Participants were then asked their vaccination status and presented with the measures of their vaccination intentions, before completing socio-demographic information and being fully debriefed.^[1]^

***Measures***

**Belief in COVID-19 vaccine conspiracy theories** (α = .94) was measured with nine items adapted from Jolley and Douglas (2014) to the COVID-19 context (e.g., “COVID-19 vaccines are harmful, and this fact is covered up”), using a response scale from 1 (*strongly disagree*) to 7 (*strongly agree*).

**Belief in plausible COVID-19 vaccine meta-conspiracy theories** (α = .89) was measured with five items capturing elements of the belief that nefarious groups secretly collude to spread false information about COVID-19 vaccines to achieve their agendas (e.g., “Secret groups who are trying to destabilise our society are spreading false information about COVID-19 vaccines”), using a response scale from 1 (*strongly disagree*) to 7 (*strongly agree*).

**COVID-19 vaccination intentions** were measured by first asking participants whether they had been vaccinated against COVID-19 (*N* = 256 fully vaccinated, *N* = 116 completely unvaccinated). If participants were completely unvaccinated, they were presented with a scale asking if they would accept a COVID-19 vaccine that inoculates against current variants of COVID-19 if offered it the following week*.* Next, these participants, and participants who selected one of the options indicating that they had been previously vaccinated against COVID-19 (*N* = 48 no second dose but intended to have it, *N* = 15 no second dose and did not intend to have it), were presented with a scale asking if they would accept a new vaccine that works against a future variant of COVID-19. In both cases, participants used a response scale from 1 (*definitely not vaccinate*) to 7 (*definitely vaccinate*).

**Socio-demographics.** Aside from their age and gender, participants indicated their highest level of completed education (*M* = 5.06, *SD* = 0.62), their levels of religiosity (*M* = 4.47, *SD* = 2.08), alongside their self-placed general (*M* = 5.81, *SD* = 2.53), social (*M* = 5.62, *SD* = 2.53), and economic political ideology on a left-right scale (*M* = 5.97, *SD* = 2.50).

**Results**

***Main analyses***

COVID-19 vaccine conspiracy beliefs had a very large positive correlation with belief in plausible COVID-19 vaccine meta-conspiracy theories, but only belief in plausible COVID-19 vaccine meta-conspiracy theories had large-to-very large positive correlations with intentions to vaccinate against both current and future variants of COVID-19 (see Table S1).

**Table S1**

*Means, Standard Deviations, and Zero-Order Correlations between the Main Variables (Pilot Study).*

| Measure | *M* | *SD* | 1 | 2 | 3 | 4 |
| --- | --- | --- | --- | --- | --- | --- |
| 1. COVID-19 vaccine conspiracy beliefs | 4.24 | 1.70 | - | .57* | -.14 | .02 |
| 2. COVID-19 vaccine meta-conspiracy beliefs | 4.68 | 1.50 |  | - | .40* | .31* |
| 3. COVID-19 vaccination intentions (current) | 4.53 | 2.11 |  |  | - | .89* |
| 4. COVID-19 vaccination intentions (future) | 5.58 | 1.57 |  |  |  | - |

Note: ⁎ *p* < .001. COVID-19 vaccination intentions (current) = intentions to vaccinate against **current** variants of COVID-19; COVID-19 vaccination intentions (future) = intentions to vaccinate against a **future** variant of COVID-19. COVID-19 vaccination intentions (current) *N* = 116; Vaccination intentions (future) *N* = 438.

When their shared variance was controlled for, intentions to vaccinate against both current and future variants of COVID-19 were negatively related to COVID-19 vaccine conspiracy beliefs and positively related to belief in plausible COVID-19 vaccine meta-conspiracy theories (see Table S2).

**Table S2**

*Regression Model with COVID-19 Vaccine Conspiracy Beliefs and COVID-19 Vaccine Meta-Conspiracy Beliefs as the Predictors, and COVID-19 Vaccination Intentions Against Both Current and Future Variants as the Dependent Variables (Pilot Study).*

|  | Vaccination intentions (current) | | | |  | Vaccination intentions (future) | | | |
| --- | --- | --- | --- | --- | --- | --- | --- | --- | --- |
| Predictor | β | *SE* | 95% CI | *p* |  | β | *SE* | 95% CI | *p* |
| Consp. | -.29 | .08 | [-.43, -.13] | < .001 |  | -.23 | .05 | [-.34, -.12] | < .001 |
| Meta. | .49 | .08 | [.32, .64] | < .001 |  | .44 | .06 | [.31, 0.57] | < .001 |
|  | *R^2^* = 0.24 | | | |  | *R^2^* = 0.13 | | | |
|  | *F*(2, 113) = 17.31* | | | |  | *F*(2, 435) = 33.68* | | | |

*Note.* **p* < .001. Consp. = COVID-19 vaccine conspiracy beliefs; Meta. = COVID-19 vaccine meta-conspiracy beliefs.

These findings remained similar for vaccination intentions against future variants of COVID-19 when additionally controlling for the shared variance with education (a common correlate of belief in COVID-19 vaccine conspiracy theories, plausible COVID-19 vaccine meta-conspiracy theories, and vaccination intentions against future variants of COVID-19; see Table S3).

**Table S3**

*Regression Model with COVID-19 Vaccine Conspiracy Beliefs, COVID-19*

*Vaccine Meta-Conspiracy Beliefs, and Education as the Predictors, and COVID-19 Vaccination Intentions Against Future Variants as the Dependent Variable (Pilot Study).*

| Predictor | β | *SE* | 95% CI | *p* |
| --- | --- | --- | --- | --- |
| Consp. | -.27 | .05 | -.37, -.16 | < .001 |
| Meta. | .45 | .06 | .32, 0.57 | < .001 |
| Education | .10 | .05 | .01, .20 | .042 |
|  | *R^2^* = 0.14 | | | |
|  | *F*(3, 434) = 24.18* | | | |

*Note.* **p* < .001. Consp. = COVID-19 vaccine conspiracy beliefs; Meta. = COVID-19 vaccine meta-conspiracy beliefs.

**Latent profile analysis**

Latent profile analysis indicated that indices were similar for both the three-profile, Entropy = 0.97, AveP_min_ = 0.97, and two-profile solutions, Entropy = 0.96, and AveP_min_ = 0.98, were equally appropriate (see Figure S1).

**Figure S1**

*Profile Plots for 1 to 5 Profiles in the Latent Profile Analysis (Pilot Study)*

**Section 2: Study 1**

**Latent profile analysis**

Latent profile analysis indicated that indices were similar for both the three-profile, Entropy = 0.99, AveP_min_ = 0.96, and two-profile solutions, Entropy = 1.00, and AveP_min_ = 1.00, were equally appropriate (see Figure S2).

**Figure S2**

*Profile Plots for 1 to 5 Profiles in the Latent Profile Analysis (Study 1)*

**Interaction effects**

Moderation analysis indicated that there was a significant interaction between belief in COVID-19 vaccine meta-conspiracies and general conspiracy beliefs when predicting COVID-19 vaccination intentions and controlling for belief in COVID-19 vaccine conspiracy theories, *F*(1, 715) = 4.63, *p* = .032. Deconstruction of this moderation effect revealed that the relationship between belief in COVID-19 vaccine meta-conspiracies and COVID-19 vaccination intentions was positive and significant at all levels of general conspiracy beliefs, but relatively weaker when general conspiracy beliefs were weaker, *B* = 0.20, *SE* = 0.04, 95% CI [0.11, 0.28], *p* < .001, stronger when general conspiracy beliefs were moderate, *B* = 0.26, *SE* = 0.03, 95% CI [0.19, 0.32], *p* < .001, and strongest when general conspiracy beliefs were stronger, *B* = 0.31, *SE* = 0.04, 95% CI [0.23, 0.39], *p* < .001 (see Figure S3).

**Figure S3**

*Simple Slopes Plot for the Interaction Between Belief in COVID-19 Vaccine Meta-Conspiracies and General Conspiracy Beliefs when Predicting COVID-19 Vaccination Intentions and Controlling for Belief in COVID-19 Vaccine Conspiracy Theories (Study 1)*

COVID-19 vaccination intentions

| Belief in COVID-19 vaccine meta-conspiracies | High general conspiracy beliefs (+1SD) | Moderate general conspiracy beliefs (Mean) | Low general conspiracy beliefs (-1SD) |
| --- | --- | --- | --- |
| Low | 5.4987 | 5.7078 | 5.9168 |
| High | 6.3295 | 6.3872 | 6.4451 |

**Experimental conditions.** Moderation analysis indicated that there were no significant interactions between the pre-bunk condition (vs. control) and powerlessness around COVID-19, *F*(1, 716) = 0.98, *p* = .323, or uncertainty around COVID-19, *F*(1, 716) = 1.50, *p* = .221, when predicting belief in COVID-19 vaccine meta-conspiracies. Furthermore, there was no significant interaction between the meta-conspiracy condition (vs. control) and powerlessness around COVID-19, *F*(1, 716) = 0.10, *p* = .757, when predicting belief in COVID-19 vaccine meta-conspiracies. However, there was a significant interaction between the meta-conspiracy condition (vs. control) and uncertainty around COVID-19 when predicting belief in COVID-19 meta-conspiracies, *F*(1, 716) = 6.17, *p* = .013. Deconstruction of this moderation effect revealed that the positive effect of the meta-conspiracy condition (vs. control) on belief in COVID-19 vaccine meta-conspiracies was significant and strongest when uncertainty was low, *B* = 0.46, *SE* = 0.11, 95% CI [0.26, 0.67], *p* < .001, weaker but still significant when uncertainty was moderate, *B* = 0.29, *SE* = 0.07, 95% CI [0.14, 0.43], *p* < .001, and non-significant when uncertainty was high, *B* = 0.11, *SE* = 0.10, 95% CI [-0.09, 0.30], *p* = .273 (see Figure S4).

**Figure S4**

*Simple Slopes Plot for the Interaction Between the Meta-Conspiracy Condition (vs. Control) and Uncertainty Around COVID-19 when Predicting Belief in COVID-19 Vaccine Meta-Conspiracies (Study 1)*

Belief in COVID-19 vaccine meta-conspiracies

| Condition | High uncertainty (+1SD) | Moderate uncertainty (Mean) | Low uncertainty (-1SD) |
| --- | --- | --- | --- |
| Control | 4.5103 | 4.8063 | 5.1024 |
| Meta-conspiracy | 4.6496 | 5.172 | 5.6945 |

**Belief in COVID-19 vaccine conspiracy theories.** Moderation analysis indicated that there were no significant interactions between belief in COVID-19 vaccine conspiracy theories and powerlessness around COVID-19, *F*(1, 716) = 0.03, *p* = .857, or uncertainty around COVID-19, *F*(1, 716) = 0.27, *p* = .601, when predicting COVID-19 vaccination intentions. However, there was a significant interaction between belief in COVID-19 vaccine conspiracy theories and age when predicting COVID-19 vaccination intentions, *F*(1, 716) = 4.37, *p* = .037. Deconstruction of this moderation effect indicated that the relationship between belief in COVID-19 vaccine conspiracy theories and COVID-19 vaccination intentions was negative and significant when age was lower, *B* = -1.89, *SE* = 0.07, 95% CI [-2.02, -1.75], *p* < .001, and weaker but still negative and significant when age was higher, *B* = -1.69, *SE* = 0.07, 95% CI [-1.82, -1.56], *p* < .001 (see Figure S5).

**Figure S5**

*Simple Slopes Plot for the Interaction Between Belief in COVID-19 Vaccine Conspiracy Theories and Age when Predicting COVID-19 Vaccination Intentions (Study 1)*

COVID-19 vaccination intentions

|  | Low age (-1SD) | High age (+1SD) |
| --- | --- | --- |
| Low belief in COVID-19 vaccine conspiracy theories (-1SD) | 6.9273 | 6.961 |
| High belief in COVID-19 vaccine conspiracy theories (+1SD) | 4.4266 | 4.7176 |

Furthermore, there was a significant interaction between belief in COVID-19 vaccine conspiracy theories and social political ideology when predicting COVID-19 vaccination intentions, *F*(1, 716) = 4.13, *p* = .008. Deconstruction of this moderation effect indicated that the relationship between belief in COVID-19 vaccine conspiracy theories and COVID-19 vaccination intentions was negative and significant among socially liberal participants, *B* = -1.89, *SE* = 0.12, 95% CI [-2.12, -1.66], *p* < .001, and weaker but still negative and significant among socially conservative participants, *B* = -1.65, *SE* = 0.06, 95% CI [-1.77, -1.54], *p* < .001 (see Figure S6).

**Figure S6**

*Simple Slopes Plot for the Interaction Between Belief in COVID-19 Vaccine Conspiracy Theories and Social Ideology when Predicting COVID-19 Vaccination Intentions (Study 1)*

COVID-19 vaccination intentions

|  | Social liberals (-1SD) | Social conservatives (+1SD) |
| --- | --- | --- |
| Low belief in COVID-19 vaccine conspiracy theories (-1SD) | 7.195 | 6.557 |
| High belief in COVID-19 vaccine conspiracy theories (+1SD) | 4.6885 | 4.3687 |

**Belief in COVID-19 vaccine meta-conspiracies.** Moderation analysis indicated that there was an interaction between belief in COVID-19 vaccine meta-conspiracies and powerlessness around COVID-19 when predicting COVID-19 vaccination intentions, *F*(1, 716) = 42.21, *p* < .001. Deconstruction of this moderation effect revealed that the relationship between belief in COVID-19 vaccine meta-conspiracies and COVID-19 vaccination intentions was positive and significant at all levels of powerlessness, but weakest when powerlessness was low, *B* = 0.33, *SE* = 0.06, 95% CI [0.21, 0.44], *p* < .001, stronger when powerlessness was moderate, *B* = 0.57, *SE* = 0.04, 95% CI [0.49, 0.66], *p* < .001, and strongest when powerlessness was high, *B* = 0.82, *SE* = 0.05, 95% CI [0.72, 0.93], *p* < .001 (see Figure S7). This interaction effect remained similar when additionally controlling for belief in COVID-19 vaccine conspiracy theories, *F*(1, 715) = 15.18, *p* < .001 (see Figure S7).

**Figure S7**

*Simple Slopes Plot for the Interaction Between Belief in COVID-19 Vaccine Meta-Conspiracies and Powerlessness Regarding COVID-19 when Predicting COVID-19 Vaccination Intentions (Study 1)*

COVID-19 vaccination intentions

| Belief in COVID-19 vaccine meta-conspiracies | High powerlessness (+1SD) | Moderate powerlessness (Mean) | Low powerlessness (-1SD) |
| --- | --- | --- | --- |
| Low (-1SD) | 4.5666 | 5.3547 | 6.1427 |
| High (+1SD) | 6.759 | 6.8844 | 7.0097 |

Moderation analysis also indicated that there was an interaction between belief in COVID-19 vaccine meta-conspiracies and uncertainty around COVID-19 when predicting COVID-19 vaccination intentions, *F*(1, 716) = 68.68, *p* < .001. Deconstruction of this moderation effect revealed that the relationship between belief in COVID-19 vaccine meta-conspiracies and COVID-19 vaccination intentions was positive and significant at all levels of uncertainty, but weakest when uncertainty was low, *B* = 0.19, *SE* = 0.06, 95% CI [0.08, 0.30], *p* < .001, stronger when uncertainty was moderate, *B* = 0.47, *SE* = 0.04, 95% CI [0.39, 0.54], *p* < .001, and strongest when uncertainty was high, *B* = 0.74, *SE* = 0.05, 95% CI [0.65, 0.84], *p* < .001 (see Figure S8). This interaction effect remained similar when additionally controlling for belief in COVID-19 vaccine conspiracy theories, *F*(1, 715) = 9.60, *p* = .002.

**Figure S8**

*Simple Slopes Plot for the Interaction Between Belief in COVID-19 Vaccine Meta-Conspiracies and Uncertainty Regarding COVID-19 when Predicting COVID-19 Vaccination Intentions (Study 1)*

COVID-19 vaccination intentions

| Belief in COVID-19 vaccine meta-conspiracies | High uncertainty (+1SD) | Moderate uncertainty (Mean) | Low uncertainty (-1SD) |
| --- | --- | --- | --- |
| Low (-1SD) | 5.4585 | 5.7795 | 6.1006 |
| High (+1SD) | 6.2718 | 6.3719 | 6.472 |

Moderation analysis also indicated that there was a significant interaction between belief in COVID-19 vaccine meta-conspiracies and gender when predicting COVID-19 vaccination intentions, *F*(1, 693) = 29.36, *p* < .001. Deconstruction of this moderation effect revealed that the relationship between belief in COVID-19 vaccine meta-conspiracies and COVID-19 vaccination intentions was positive and significant among women, *B* = 0.85, *SE* = 0.05, 95% CI [0.74, 0.95], *p* < .001, and weaker but still positive and significant among men, *B* = 0.36, *SE* = 0.07, 95% CI [0.22, 0.50], *p* < .001 (see Figure S9).

**Figure S9**

*Simple Slopes Plot for the Interaction Between Belief in COVID-19 Vaccine Meta-Conspiracies and Gender when Predicting COVID-19 Vaccination Intentions (Study 1)*

COVID-19 vaccination intentions

|  | Men | Women |
| --- | --- | --- |
| Low belief in COVID-19 vaccine meta-conspiracies (-1SD) | 5.9108 | 4.6918 |
| High belief in COVID-19 vaccine meta-conspiracies (+1SD) | 6.8624 | 6.9462 |

Moderation analysis also indicated that there was a significant interaction between belief in COVID-19 vaccine meta-conspiracies and religiosity when predicting COVID-19 vaccination intentions, *F*(1, 716) = 8.25, *p* = .004. Deconstruction of this moderation effect revealed that the relationship between belief in COVID-19 vaccine meta-conspiracies and COVID-19 vaccination intentions was positive and significant when participants were more religious, *B* = 1.60, *SE* = 0.05, 95% CI [0.62, 0.84], *p* < .001, and weaker but still positive and significant when participants were less religious, *B* = 0.55, *SE* = 0.05, 95% CI [0.44, 0.65], *p* < .001 (see Figure S10).

**Figure S10**

*Simple Slopes Plot for the Interaction Between Belief in COVID-19 Vaccine Meta-Conspiracies and Religiosity when Predicting COVID-19 Vaccination Intentions (Study 1)*

COVID-19 vaccination intentions

|  | High religiosity (+1SD) | Low religiosity (-1SD) |
| --- | --- | --- |
| Low belief in COVID-19 vaccine meta-conspiracies (-1SD) | 4.8367 | 5.5185 |
| High belief in COVID-19 vaccine meta-conspiracies (+1SD) | 6.7862 | 6.9756 |

Moderation analysis also indicated that there was a significant interaction between belief in COVID-19 vaccine meta-conspiracies and general political ideology when predicting COVID-19 vaccination intentions, *F*(1, 716) = 71.21, *p* < .001. Deconstruction of this moderation effect revealed that the relationship between belief in COVID-19 vaccine meta-conspiracies and COVID-19 vaccination intentions was positive and significant among generally conservative participants, *B* = 0.66, *SE* = 0.05, 95% CI [0.57, 0.76], *p* < .001, and generally centrist participants, *B* = 0.35, *SE* = 0.04, 95% CI [0.27, 0.44], *p* < .001, but not among generally liberal participants, *B* = 0.04, *SE* = 0.06, 95% CI [-0.09, 0.17], *p* = .543 (see Figure S11).

**Figure S11**

*Simple Slopes Plot for the Interaction Between Belief in COVID-19 Vaccine Meta-Conspiracies and General Political Ideology when Predicting COVID-19 Vaccination Intentions (Study 1)*

COVID-19 vaccination intentions

|  | General conservatives (+1SD) | General centrists (Mean) | General liberals (-1SD) |
| --- | --- | --- | --- |
| Low belief in COVID-19 vaccine meta-conspiracies (-1SD) | 4.7001 | 5.7389 | 6.7777 |
| High belief in COVID-19 vaccine meta-conspiracies (+1SD) | 6.4689 | 6.6757 | 6.8824 |

Moderation analysis also indicated that there was a significant interaction between belief in COVID-19 vaccine meta-conspiracies and social political ideology when predicting COVID-19 vaccination intentions, *F*(1, 716) = 57.22, *p* < .001. Deconstruction of this moderation effect revealed that the relationship between belief in COVID-19 vaccine meta-conspiracies and COVID-19 vaccination intentions was positive and significant among socially conservative participants, *B* = 0.66, *SE* = 0.05, 95% CI [0.57, 0.76], *p* < .001, socially centrist participants, *B* = 0.40, *SE* = 0.04, 95% CI [0.32, 0.48], *p* < .001, and weaker but still positive and significant among socially liberal participants, *B* = 0.13, *SE* = 0.06, 95% CI [0.01, 0.25], *p* = .031 (see Figure S12).

**Figure S12**

*Simple Slopes Plot for the Interaction Between Belief in COVID-19 Vaccine Meta-Conspiracies and Social Political Ideology when Predicting COVID-19 Vaccination Intentions (Study 1)*

COVID-19 vaccination intentions

|  | Social conservatives (+1SD) | Social centrists (Mean) | Social liberals (-1SD) |
| --- | --- | --- | --- |
| Low belief in COVID-19 vaccine meta-conspiracies (-1SD) | 4.6855 | 5.6403 | 6.5951 |
| High belief in COVID-19 vaccine meta-conspiracies (+1SD) | 6.4533 | 6.7018 | 6.9504 |

Moderation analysis also indicated that there was a significant interaction between belief in COVID-19 vaccine meta-conspiracies and economic political ideology when predicting COVID-19 vaccination intentions, *F*(1, 716) = 78.87, *p* < .001. Deconstruction of this moderation effect revealed that the relationship between belief in COVID-19 vaccine meta-conspiracies and COVID-19 vaccination intentions was positive and significant among economically conservative participant, *B* = 0.75, *SE* = 0.05, 95% CI [0.66, 0.85], *p* < .001, and economically centrist participants, *B* = 0.42, *SE* = 0.04, 95% CI [0.34, 0.51], *p* < .001, but non-significant among economically liberal participants, *B* = 0.09, *SE* = 0.06, 95% CI [-0.03, 0.21], *p* = .146 (see Figure S13).

**Figure S13**

*Simple Slopes Plot for the Interaction Between Belief in COVID-19 Vaccine Meta-Conspiracies and Economic Political Ideology when Predicting COVID-19 Vaccination Intentions (Study 1)*

COVID-19 vaccination intentions

|  | Economic conservatives (+1SD) | Economic centrists (Mean) | Economic liberals (-1SD) |
| --- | --- | --- | --- |
| Low belief in COVID-19 vaccine meta-conspiracies (-1SD) | 4.6072 | 5.6267 | 6.6462 |
| High belief in COVID-19 vaccine meta-conspiracies (+1SD) | 6.6172 | 6.7532 | 6.8892 |

**Indirect path model**

There were no significant total effects of the prebunk (vs. control), β = .08, *p* = .075, meta-conspiracy (vs. control), β = .04, *p* = .190, debunk (vs. control), β = -.03, *p* = .496, or conspiracy theories (vs. control) conditions, β = -.01, *p* = .777, on COVID-19 vaccination intentions. Furthermore, there were no indirect effects of the debunk (vs. control), standardized indirect effect = .01, 95% CI [-.01, .02], or conspiracy theories (vs. control) conditions, standardized indirect effect = -.01, 95% CI [-.03, .01], on COVID-19 vaccination intentions through belief in COVID-19 vaccine meta-conspiracy theories. There were also no indirect effects of the prebunk (vs. control), standardized indirect effect = .05, 95% CI [-.01, .10], meta-conspiracy (vs. control), standardized indirect effect = .03, 95% CI [-.03, .08], debunk (vs. control), standardized indirect effect = -.03, 95% CI [-.08, .03], or conspiracy theories (vs. control) conditions, standardized indirect effect = -.01, 95% CI [-.06, .05], on COVID-19 vaccination intentions though belief in COVID-19 vaccine conspiracy theories.

**Experimental manipulations**

***Meta-conspiracy theories***

**Please read this short excerpt from a recent Internet article about COVID-19 vaccines. We will ask you some questions about the excerpt later in the study, so please read it carefully.**

**Note:** You will not be able to skip to the next page until at least 30 seconds have passed (see countdown clock below).

Should we be suspicious of people who spread false information about COVID-19 vaccines? Should we consider the proposal that those who spread this information, whether governments or alternative-medicine moguls, do so deliberately for their own personal gain?

There are good reasons to be suspicious. For example, a recent investigation found that Russian, Chinese, and Iranian intelligence agencies colluded to spread false information about COVID-19 vaccines. Servers were seized from a recently abandoned warehouse in Uzbekistan, detailing plans for how and where online “trolls” should spread false information. This included the idea that Bill Gates was using the COVID-19 vaccine to track citizens, and fake statistics about harmful vaccine side-effects. A recent report concluded that these agencies colluded to spread this false information to disempower the west and protect their own countries’ interests.

Because of this false information, it is not surprising that people are worried. Polls have reported that around one fifth of the population have hesitated to have the COVID-19 vaccine.

***Conspiracy theories***

**Please read this short excerpt from a recent Internet article about COVID-19 vaccines. We will ask you some questions about the excerpt later in the study, so please read it carefully.**

**Note:** You will not be able to skip to the next page until at least 30 seconds have passed (see countdown clock below).

Should we be suspicious of COVID-19 vaccines? Should we consider the proposal that those in power, whether governments or pharmaceutical companies, hide crucial information about COVID-19 vaccines from the public?

There are good reasons to be suspicious. For example, online videos of people having the COVID-19 vaccine often look fake. Further, there has been a worrying number of deaths and health complications as a direct result of the COVID-19 vaccinations and these deaths have been covered up as being deaths from other causes. There are other suspicious circumstances too. For example, the media focuses disproportionately on the complications caused by the virus to try and coerce the public to accept COVID-19 vaccinations. Finally, governments have exaggerated the dangers of the virus so that they can push through specific draconian measures such as mandatory COVID-19 vaccination.

Because of these suspicious circumstances, it is not surprising that people are worried. Polls have reported that around one fifth of the population have hesitated to have the COVID-19 vaccine.

***Control condition***

When you're ready, click the arrow on the bottom right to begin the study.

**Scale items**

***Belief in COVID-19 vaccine meta-conspiracy theories***

1. Alternative medicine moguls are spreading false information about Covid-19 vaccines in order to promote their alternative treatments.
2. Secret groups who are trying to destabilise our society are spreading false information about Covid-19 vaccines.
3. Covert groups are purposely sowing doubt about Covid-19 vaccines in the public by spreading false information about them.
4. Malign foreign governments are actively trying to harm our health and economic recovery by spreading false information about Covid-19 vaccines.
5. False information about Covid-19 vaccines is created maliciously, with the intention of purposely misleading the public about them in order to deter people from being vaccinated.

***Belief in COVID-19 vaccine conspiracy theories***

1. Bad actors in the government and pharmaceutical companies are secretly working together to cover up the truth about Covid vaccines.
2. Covid-19 vaccinations allow governments to track and control people.
3. Covid-19 vaccines are harmful, and this fact is covered up.
4. Tiny devices are placed in Covid-19 vaccines to track people.
5. Pharmaceutical companies, scientists and academics work together to cover up the dangers of Covid-19 vaccines.
6. Covid-19 vaccines are not tampered with (reverse-coded).
7. The government is trying to cover up the link between Covid-19 vaccines and autism.
8. Tiny devices are implanted in Covid-19 vaccines for use in mind control experiments.
9. The Covid-19 vaccines allow the government to monitor the elderly through the implantation of tiny tracking devices.
10. Please select the seventh option "strongly agree" to indicate that you are paying attention.

***Vaccination intentions***

*Imagine for a moment that a new variant of COVID-19 is in circulation and it is fully resistant to our current vaccines. Some time has passed and scientists have been able to create new vaccines that work against this new variant.*

*If you had the opportunity to vaccinate yourself against this new variant of COVID-19 next week, what would you decide?*

[1 = Definitely not vaccine; 7 = Definitely vaccinate.]

***Conspiracy mentality***

*I think that...*

1. …many very important things happen in the world, which the public is never informed about.
2. …politicians usually do not tell us the true motives for their decisions.
3. …government agencies closely monitor all citizens.
4. …events which superficially seem to lack a connection are often the result of secret activities.
5. …there are secret organizations that greatly influence political decisions.

***Powerlessness around COVID-19***

1. I feel that the COVID-19 pandemic is too big for my actions to have an impact.
2. I feel that my actions will not affect the outcome the COVID-19 pandemic.
3. I feel that my contribution is just a drop in the ocean and so is insignificant.

***Uncertainty around COVID-19***

1. I feel uncertain about the origin of COVID-19.
2. I feel uncertain about the dangers of COVID-19.
3. I feel uncertain about the scientific advice surrounding COVID-19.

**Section 3: Study 2**

**Pre-screen checks**

***Prior conspiracy beliefs***

A one-way ANOVA, *F(*2, 1074) = 71.77, *p* < .001, with a Tukey post-hoc correction indicated that general conspiracy beliefs were significantly higher among climate deniers than both climate skeptics, *p* < .001, *d* = 0.48, and climate accepters, *p* < .001, *d* = 0.76 (see Figure S14). However, there was no significant difference in general conspiracy beliefs between climate skeptics and climate accepters, *p* = .055, *d* = 0.28.

**Figure S14**

*Violin Plot for the Mean Difference in Prior Conspiracy Beliefs between Pre-Screened Climate Deniers, Climate Skeptics, and Climate Accepters (Study 2)*

***Belief in anthropogenic climate change***

A one-way ANOVA, *F(*2, 1074) = 853, *p* < .001, with a Tukey post-hoc correction indicated that belief in anthropogenic climate change was significantly lower among climate deniers than both climate skeptics, *p* < .001, *d* = 0.96, and climate accepters, *p* < .001, *d* = 2.61 (see Figure S15). Furthermore, belief in anthropogenic climate change was significantly lower among climate skeptics than climate accepters, *p* < .001, *d* = 1.65.

**Figure S15**

*Violin Plot for the Mean Difference in Belief in Anthropogenic Climate Change between Pre-Screened Climate Deniers, Climate Skeptics, and Climate Accepters (Study 2)*

**Regression analyses**

**Table S4**

*Bootstrapped* *Regression Coefficients, Standard Errors, 95% Confidence Intervals, and P-Values for the Predictors of Climate Intentions (Study 3).*

| Predictor | β | *SE* | 95% CI | *p* |
| --- | --- | --- | --- | --- |
| Belief in COVID-19 vaccine meta-conspiracies | .17 | .03 | .11, 0.23 | < .001 |
| Belief in COVID-19 vaccine conspiracy theories | -.42 | .04 | -.50, -.34 | < .001 |
| General conspiracy beliefs | .06 | .03 | .01, .12 | .032 |
| Perceived scientific consensus | -.01 | .03 | -.07, .05 | .702 |
| Religiosity | .15 | .03 | .09, .20 | < .001 |
| General conservatism | -.22 | .11 | -.42, -.01 | .045 |
| Social conservatism | -.01 | .08 | -.16, .15 | .950 |
| Economic conservatism | .03 | .07 | -.10, .17 | .646 |
|  | *R^2^* = 0.34 | | | |
|  | *F*(8, 1067) = 69.72* | | | |

*Note.* * *p* < .001.

**Interaction effects**

***Fighting fire with fire prebunk vs. control.*** Moderation analysis indicated that there were no significant interaction effects between the fighting with fire prebunk (vs. control) and general conspiracy beliefs, *F*(1, 537) = 1.45, *p* = .229, *R^2^* = 0.02, pre-screened climate acceptance, *F*(1, 537) = 0.94, *p* = .334, *R^2^* = 0.14, or anthropogenic climate change, *F*(1, 537) = 2.36, *p* = .125, *R^2^* = 0.22, when predicting belief in plausible climate change meta-conspiracy theories. However, there was a significant interaction effect between the fighting fire with fire prebunk (vs. control) and economic political ideology when predicting belief in plausible climate change meta-conspiracy theories, *F*(1, 537) = 4.67, *p* = .031, *R^2^* = 0.14, such that the positive effect of the fighting fire with fire prebunk (vs. control) on belief in plausible climate change meta-conspiracy theories was only significant among participants who were more economically liberal, *B* = 0.45, *SE* = 0.11, 95% CI [0.24, 0.67], *p* < .001, and economically centrist, *B* = 0.24, *SE* = 0.06, 95% CI [0.11, 0.36], *p* < .001, but not among participants who were more economically conservative, *B* = 0.13, *SE* = 0.09, 95% CI [-0.04, 0.30], *p* = .126 (see Figure S16).

**Figure S16**

*Simple Slopes Plot for the Interaction Between the Fighting Fire with Fire Prebunk (vs. Control) and Economic Political Ideology when Predicting Belief in Climate Change Meta-Conspiracies (Study 2)*

***Standard prebunk vs. control.*** There was a significant interaction between the standard prebunk (vs. control) and general conspiracy beliefs when predicting belief in plausible climate change meta-conspiracy theories, *F*(1, 531) = 9.24, *p* = .003, *R^2^* = 0.04, such that the positive effect of the standard prebunk (vs. control) on belief in plausible climate change meta-conspiracy theories was only significant when general conspiracy beliefs were weaker, *B* = 0.50, *SE* = 0.09, 95% CI [0.32, 0.68], *p* < .001, or moderate, *B* = 0.27, *SE* = 0.06, 95% CI [0.15, 0.39], *p* < .001, but not when general conspiracy beliefs were stronger, *B* = 0.04, *SE* = 0.10, 95% CI [-0.16, 0.24], *p* = .700 (see Figure S17).

**Figure S17**

*Simple Slopes Plot for the Interaction Between the Standard Prebunk (vs. Control) and General Conspiracy Beliefs when Predicting Belief in Climate Change Meta-Conspiracies (Study 2)*

There was also a significant interaction between the standard prebunk (vs. control) and the pre-screened sub-samples when predicting belief in plausible climate change meta-conspiracy theories, *F*(1, 531) = 6.20, *p* = .013, *R^2^* = 0.18, such that the positive effect of the standard prebunk (vs. control) on belief in plausible climate change meta-conspiracy theories was only significant among climate accepters, *B* = 0.43, *SE* = 0.09, 95% CI [0.26, 0.60], *p* < .001, and climate skeptics, *B* = 0.35, *SE* = 0.07, 95% CI [0.21, 0.48], *p* < .001, but not among climate deniers, *B* = 0.11, *SE* = 0.09, 95% CI [-0.08, 0.29], *p* = .251 (see Figure S18).

**Figure S18**

*Simple Slopes Plot for the Interaction Between the Standard Prebunk (vs. Control) and the Pre-Screened Sub-Samples when Predicting Belief in Climate Change Meta-Conspiracies (Study 2)*

There was also a significant interaction between the standard prebunk (vs. control) and belief in anthropogenic climate change when predicting belief in plausible climate change meta-conspiracy theories, *F*(1, 531) = 9.16, *p* = .003, *R^2^* = 0.27, such that the positive effect of the standard prebunk (vs. control) on belief in plausible climate change meta-conspiracy theories was only significant when belief in anthropogenic climate change was stronger, *B* = 0.46, *SE* = 0.09, 95% CI [0.27, 0.64], *p* < .001, or moderate, *B* = 0.24, *SE* = 0.06, 95% CI [0.13, 0.36], *p* < .001, but not when belief in anthropogenic climate change was weaker, *B* = 0.03, *SE* = 0.09, 95% CI [-0.15, 0.21], *p* = .767 (see Figure S19).

**Figure S19**

*Simple Slopes Plot for the Interaction Between the Standard Prebunk (vs. Control) and Belief in Anthropogenic Climate Change when Predicting Belief in Climate Change Meta-Conspiracies (Study 2)*

***Conspiracy vs. control.*** There was a significant interaction between the conspiracy condition (vs. control) and religiosity when predicting belief in plausible climate change meta-conspiracy theories, *F*(1, 553) = 5.65, *p* = .018, *R^2^* = 0.05, such that the positive effect of the conspiracy condition (vs. control) on belief in plausible climate change meta-conspiracy theories was only significant when religiosity was weaker, *B* = 0.35, *SE* = 0.10, 95% CI [0.16, 0.55], *p* < .001, and moderate, *B* = 0.14, *SE* = 0.07, 95% CI [0.01, 0.27], *p* = .035, but not when religiosity was stronger, *B* = 0.01, *SE* = 0.10, 95% CI [-0.19, 0.19], *p* = .988 (see Figure S20).

**Figure S20**

*Simple Slopes Plot for the Interaction Between the Conspiracy Condition (vs. Control) and Religiosity when Predicting Belief in Climate Change Meta-Conspiracies (Study 2)*

***Education***

There was a significant interaction between the standard pre-bunk (vs. control) and education, *F*(1, 530) = 5.82, *p* = .016, such that the positive effect of the standard pre-bunk (vs. control) on belief in climate change meta-conspiracies was strongest and significant when education levels were low, *B* = 0.44, *SE* = 0.09, 95% CI [0.26, 0.61], *p* < .001, weaker but still significant when education levels were moderate, *B* = 0.28, *SE* = 0.06, 95% CI [0.16, 0.41], *p* < .001, and non-significant when education levels were high, *B* = 0.13, *SE* = 0.09, 95% CI [-0.04, 0.31], *p* = .135 (see Figure S21).

**Figure S21**

*Simple Slopes Plot for the Interaction Between the Standard Pre-Bunk (vs. Control) and Education Levels when Predicting Belief in Climate Change Meta-Conspiracies (Study 2)*

Belief in climate change meta-conspiracies

| Condition | High education levels (+1SD) | Moderate education levels (average) | Low education levels (-1SD) |
| --- | --- | --- | --- |
| Control | 3.7999 | 3.6408 | 3.4818 |
| Standard pre-bunk | 4.0663 | 4.2107 | 4.3551 |

**Latent profile analysis**

Latent profile analysis indicated that indices were most appropriate for the two-profile solution, Entropy = 0.98, AveP_min_ = 0.99 (see Figure S22).

**Figure S22**

*Profile Plots for 1, 2, and 4 Profiles in the Latent Profile Analysis (Study 2)*

**Indirect path model**

There were no significant total effects of the fighting fire with fire prebunk (vs. control), β = .01, *p* = .876, standard prebunk (vs. control), β = .09, *p* = .184, or exposure to climate change conspiracy theories (vs. control) conditions, β = -.01, *p* = .933, on climate intentions. Furthermore, there were no indirect effects of the fighting fire with fire prebunk (vs. control) on climate intentions through either belief in climate change conspiracy theories, standardized indirect effect = -.05, 95% CI [-.12, .01], or perceived scientific consensus around climate climate change, standardized indirect effect = .00, 95% CI [-.01, .01]. Similarly, there were no indirect effects of the standard prebunk (vs. control) on climate intentions through either belief in climate change conspiracy theories, standardized indirect effect = -.05, 95% CI [-.11, .02], or perceived scientific consensus around climate change, standardized indirect effect = -.01, 95% CI [-.02, .01]. Finally, there were no indirect effects of the exposure to climate change conspiracy theories condition (vs. control) on climate intentions through either belief in climate change conspiracy theories, standardized indirect effect = -.05, 95% CI [-.11, .02], or perceived scientific consensus around climate change, standardized indirect effect = .00, 95% CI [-.01, .01].

**Experimental manipulations**

***Fighting fire with fire prebunk***

**Please read this short excerpt from a recent Internet article about climate change. We will ask you some questions about the excerpt later in the study, so please read it carefully.**

**Note:** You will not be able to skip to the next page until at least 45 seconds have passed (see countdown clock above).

We should remain suspicious of people who spread false information about climate science. Those who spread this information, whether governments or oil and gas companies, may do so deliberately to downplay the dangers of climate change for their own financial or personal gain.

There are good reasons to remain vigilant. For example, a recent independent investigation found that the Koch brothers–owners of the largest conglomerate of fossil fuel companies–secretly funded and colluded with certain news companies to promote false claims that human-made climate change is not real. Furthermore, they used these outlets to deflect from the harm that their non-renewable energy extraction causes for the environment, sometimes through the dissemination of climate change conspiracy theories. Many foreign governments and oil companies are now facing an unprecedented wave of lawsuits for “the most consequential cover-up in US history” aimed to maximise profits.

Because of this secret collusion to spread false information behind the scenes that calls overwhelming scientific consensus about human-made climate change into question, it is not surprising that people are unfortunately skeptical of the existence of human-made climate change. Polls have reported that around one fifth of the population are unwilling to make changes to how they live and work to help reduce the effects of climate change.

Next time you see information questioning the existence of human-made climate change, remember to question whether the real conspiracy comes from those promoting the spread of conspiracy theories. Stay on the lookout for those with financial interests secretly colluding to call scientific consensus into question. Follow the money!

***Standard prebunk***

**Please read this short excerpt from a recent Internet article about climate change. We will ask you some questions about the excerpt later in the study, so please read it carefully.**

**Note:** You will not be able to skip to the next page until at least 45 seconds have passed (see countdown clock above).

Fossil fuel companies and political groups try to cast doubt on climate science, in order to delay regulation of the fossil fuel industry. They do this by spreading conspiracy theories calling the existance of human-made climate change into question.

Climate change conspiracy theories tend to claim that scientists exaggerate their research findings to maintain research funding and achieve prestige or status. Similar strategies were pioneered by the tobacco industry in the 1970s. They used doctors and scientists to reassure the public that smoking didn’t cause health problems. These spokespeople were either non-experts or among the small minority of scientists who dissented from the scientific consensus that smoking is bad for you. However, in the public’s eye, this conveyed the appearance of ongoing scientific debate.

The “Tobacco Strategy” is being used again, but this time to cast doubt on climate science. Less than 3% of climate scientists disagree with the consensus position that humans are causing global warming. However, when the media present the views of a dissenting scientist alongside a mainstream scientist, the public comes away with the mistaken impression of a 50:50 debate. Ironically, the journalistic standard of giving both sides equal weight has ended up distorting the state of climate science.

***Conspiracy theories***

**Please read this short excerpt from a recent Internet article about climate change. We will ask you some questions about the excerpt later in the study, so please read it carefully.**

**Note:** You will not be able to skip to the next page until at least 45 seconds have passed (see countdown clock above).
 
We should be suspicious of the supposed scientific consensus behind climate change. We should also be aware that those in power or positions of influence, whether governments or scientists, manipulate information about climate change and lie to the public.

There are good reasons to be suspicious. For example, politicians often exaggerate research findings about climate change, or they talk about research findings that are outright false. Indeed, many prominent thinkers have suggested that policies concerning climate change are just another means to exert control over people and increase global governance.

There are other suspicious circumstances too. For example, it has been shown that climate scientists have manipulated data to make climate change seem worse than it is. It is argued that they have done so in an effort to keep their research funding programme going. Furthermore, governments collude with climate scientists to perpetuate these lies.

Because of these suspicious circumstances, it is not surprising that people are skeptical about climate science. Polls have reported that around one fifth of the population are unwilling to make changes to how they live and work to help reduce the effects of climate change.

***Control condition***

**Please read this short excerpt from a recent Internet article about climate change. We will ask you some questions about the excerpt later in the study, so please read it carefully.**

**Note:** You will not be able to skip to the next page until at least 30 seconds have passed (see countdown clock above).

Ice cores provide key information about what the Earth’s climate was like in the past. Bubbles sealed in the ice provide a unique snapshot of the atmosphere at the time.

Scientists can also work out what surface temperatures were like in the past by analysing the ratio of ‘heavy’ atoms of oxygen and hydrogen. The deeper you dig, the further back in time you go. So, when a team of French and former Soviet scientists extracted an Antarctic ice core over 2,000 metres long in 1985, they were able to tell what atmospheric conditions were like 150,000 years ago. 

In 1998 the team extracted an even longer ice core, extending the climate record back to 420,000 years ago. In 2004, ice core scientists at [British Antarctic Survey](https://www.bas.ac.uk/) (BAS) were part of a team that extracted a three-kilometre ice core from the Antarctic. This core contains a record of the Earth’s climate stretching back 800,000 years, giving us by far the oldest continuous climate record yet obtained from ice cores.

**Scale items**

***Belief in climate change meta-conspiracy theories***

1. Oil and gas companies spread climate change conspiracy theories to cast doubt on human-made climate change and boost their profits.
2. Secret groups aim to destabilize society to serve their own agendas by disseminating conspiracy theories challenging the scientific consensus on climate change.
3. Covert groups intentionally sow public doubt on human-made climate change by creating and spreading climate change conspiracy theories.
4. Foreign governments purposefully undermine climate change mitigation by spreading conspiracy theories denying the harmful effects of greenhouse gases.
5. Climate change conspiracy theories are created maliciously to undermine the credibility of climate science.

***Belief in climate change conspiracy theories***

1. Climate change is a hoax.
2. Climate change has been made up by climate researchers to chase funding.
3. The official United Nations reports about climate are deliberately inaccurate.
4. Scientists are creating panic about climate change because it is in their interests to do so.
5. "Climate change" is a myth promoted by the government as an excuse to raise taxes and curb people's freedom.
6. The "science" behind climate change is at least dubious.
7. The idea that the world is headed for catastrophic climate change is a fraud.

***Perceptions of scientific consensus around climate change***

*Please estimate the percentage of climate scientists who agree that climate change is caused by human activity (e.g., burning fossil fuels).*

[0% to 100%].

***Climate intentions***

1. Use energy-efficiency as a selection criterion when buying things such as light bulbs, household appliances, or motor vehicles.
2. Explore purchasing energy from an alternative source (e.g., wind, solar, geothermal, or biomass).
3. Walk or cycle more than driving or using public transport.
4. Plant a tree.
5. Join, donate money to, or volunteer with an organization working on issues related to global warming.
6. Make your views on global warming clear to politicians.
7. Talk to family, friends, or colleagues about ways that individuals could help solve the problem of global warning.

***General conspiracy beliefs***

*Some political and social events are debated (for example 9/11 attacks, the death of Lady Diana, the assassination of John F. Kennedy). It is suggested that the “official version” of these events could be an attempt to hide the truth from the public.
This “official version” could mask the fact that these events have been planned and secretly prepared by a covert alliance of powerful individuals or organizations (for example secret services or government).
What do you think?*

*To answer, please indicate to what extent the sentence below represents how you think about this: I think that the official version of the events given by the authorities very often hides the truth.*

[1 = Completely false; 9 = Completely true].

***Acceptance of anthropogenic climate change***

1. I believe that the climate is always changing and what we are currently observing is just natural fluctuation.
2. I believe that most of the warming over the last 50 years is due to the increase in greenhouse gas concentrations.
3. I believe that the burning of fossil fuels over the last 50 years has caused serious damage to the planet’s climate.
4. Human CO2 emissions cause climate change.
5. Humans are too insignificant to have an appreciable impact on global temperature.

**References**

Douglas, K. M., & Sutton, R. M. (2015). Climate change: Why the conspiracy theories are dangerous. *Bulletin of the Atomic Scientists*, *71*(2), 98-106. <https://doi.org/10.1177/0096340215571908>

BLINDED

Jolley, D., & Douglas, K. M. (2014). The effects of anti-vaccine conspiracy theories on vaccination intentions. *PLoS ONE*, *9*(2), e89177. https://doi.org/10.1371/journal.pone.0089177

1. Other measures were presented before the conspiracy beliefs measures for the purposes of another project (see BLINDED).
